# The E3 ligase TRIM26 suppresses ferroptosis through catalyzing K63-linked ubiquitination of GPX4 in glioma

**DOI:** 10.1038/s41419-023-06222-z

**Published:** 2023-10-23

**Authors:** Zhangjie Wang, Yuan Xia, Yang Wang, Ruiqiu Zhu, Hongbo Li, Yu Liu, Na Shen

**Affiliations:** 1 Department of Neurosurgery, the First Affiliated Hospital of Nanjing Medical University, Nanjing, 210029 China; 2 Department of Hematology, the First Affiliated Hospital of Nanjing Medical University, Nanjing, 210029 China; 3 Department of Hematology, The Affiliated Taizhou People’s Hospital of Nanjing Medical University, Taizhou School of Clinical Medicine, Nanjing Medical University, Taizhou, 225300 China; 4 Department of Radiotherapy and Oncology, The Second Affiliated Hospital of Soochow University, Suzhou, 215004 China; 5 Department of Gastrointestinal Surgery, the Second Affiliated Hospital of Zhejiang University School of Medicine, Hangzhou, 310000 China

**Keywords:** Necroptosis, Ubiquitylation

## Abstract

The selenium-containing enzyme GPX4 moonlights as a central regulator of ferroptosis, an iron-dependent, nonapoptotic form of regulated cell death caused by lipid peroxidation. Yet, little is known about the mechanisms underlying the regulation of its post-transcriptional modifications. Here, we identify the tripartite motif-containing protein TRIM26 as an E3 ubiquitin ligase of GPX4. TRIM26 directly interacts with GPX4 through its Ring domain and catalyzes the ubiquitination of GPX4 at K107 and K117, which promotes the switch in polyubiquitination of GPX4 from K48 to K63, thus enhancing GPX4 protein stability. Moreover, PLK1-mediated S127 phosphorylation of TRIM26 enhances the interaction between TRIM26 and GPX4. Inhibition of TRIM26 phosphorylation causes a reduction in GPX4 K63-linked polyubiquitination and diminishes GPX4 protein levels in tumor cells. Further investigation revealed that TRIM26 is overexpressed in glioma cells. TRIM26 silencing dramatically impedes ferroptosis resistance and tumorigenesis in glioma in vivo and in vitro. Clinically, TRIM26 expression shows a direct correlation with GPX4 and PLK1 levels in glioma samples and is associated with poor outcome in patients with glioma. Collectively, these findings define the role of GPX4 K63-linked polyubiquitination in ferroptosis and suggest a potential strategy for glioma treatment.

## Introduction

Ferroptosis, a non-apoptotic form of regulated cell death driven by toxic accumulation of lipid peroxides on cellular membranes [1], has been widely implicated in the pathophysiological processes of many diseases including cancers [2–4]. GPX4, one of the eight glutathione peroxidases (GPXs), functions as a core regulator of ferroptosis by detoxifying cellular lipid peroxidation [5]. Accumulating evidence has shown that the inhibition of GPX4-mediated ferroptosis is a promising strategy for cancer therapy [6–8]. Although the essential role of GPX4 in ferroptosis has been well established, the mechanisms underlying its post-transcriptional modifications (PTMs) remain poorly understood.

TRIM26 is a member of the tripartite motif (TRIM) family proteins [9], possessing a conserved structural arrangement of the RING domain, the B-box domain and the coiled-coil motif at the N-terminus [10], which is involved in multiple biological processes, including innate immunity [11], cell proliferation [12], and the inflammatory response [13]. As an E3 ligase, TRIM26 was reported to catalyze ubiquitination of its target proteins, such as TAF7 [14], TAB1 [13], ZEB1 [12], SOX2 [15] and PBX1 [16]. Dysregulation of TRIM26 has been implicated in several cancers including glioma [15], but the molecular mechanisms governing its activation remain to be explored.

Ubiquitination, a bioprocess in which ubiquitin is covalently assembled [17], is one of the most extensively studied PTMs. It is widely implicated in the regulation of protein-protein interactions, trafficking, protein stabilization, and enzymatic activities, thereby governing a wide spectrum of cellular processes [18–20]. Accumulating evidence indicates that ubiquitin system enzymes regulate the susceptibility of cancer cells to ferroptosis [21], mainly by regulating ubiquitination and the protein stability of key regulators of ferroptosis [22]. Therefore, a more comprehensive understanding of this key modification involved in the regulation of ferroptosis may provide new insights into cancer therapeutics.

In this study, we found that GPX4 undergoes TRIM26-mediated K63-linked polyubiquitination, which promotes GPX4 protein stability by antagonizing its K48-linked polyubiquitination. Moreover, S127 phosphorylation of TRIM26 induced by PLK1 is required for the interaction between GPX4 and TRIM26. Thus, these findings provide novel insights into the regulation of GPX4 and TRIM26.

## Methods

### Cell culture

Normal human astrocytes (NHAs), human embryonic kidney cells (HEK293T) and glioma cell lines (Sw1783, Hs683, T98G, U118, A172, LN229, U251) were obtained from the ATCC. Tumor cells and HEK293T cells were maintained in DMEM supplemented with 10% fetal bovine serum and 1% penicillin/streptomycin. NHAs were maintained in AM Astrocyle Medium (1801, ScienCell). All cells were kept in a humidified incubator at 5% CO_2_ and 37 °C. The MycoAlertTM Mycoplasma Detection Kit was used to verify the negativity of mycoplasma contamination in all cell lines.

### Human glioma tissues and immunohistochemical (IHC) staining

IHC analysis was performed by Servicebio Technology (Wuhan, China) with the indicated antibodies. Staining performance was quantified by processing images using Case viewer software and 3DHISTECH QuantCenter 2.1 software. Mouse tumor tissues were fixed and prepared before staining. IHC staining intensity scores were as follows: 0, negative; 1, weak staining; 2, moderate staining; 3, strong staining; positively stained cell proportions were indicated as 0% = 0, 1%–24% = 1, 25%–49% = 2, 50%–74% = 3 and 75%–100% = 4. IHC scores were determined by multiplying the stain intensity scores by the positively stained cell proportion scores (0–12).

### Antibodies and reagents

Antibodies and reagents were acquired from designated suppliers:

TRIM26 (ab188017, Abcam), TRIM26 (27013-1-AP, Proteintech), TRIM26 (sc-393832, Santa Cruz Biotechnology), GPX4 (ab125066, Abcam), GPX4 (sc-166570, Santa Cruz Biotechnology), PLK1 (ab189139, Abcam), p-PLK1 (ab155095, Abcam), β-actin (ab8226, Abcam), K48-ubiquitin (ab140601, Abcam), K63-ubiquitin (12930, Cell Signaling Technology), anti-Flag (66008, Proteintech), anti-Myc (16286, Proteintech), p-S/T (61 G, abmart), p-S/T (612549, BD Biosciences), anti-His (66005, Proteintech), anti-Flag (14793, Cell Signaling Technology), anti-Myc (2276, Cell Signaling Technology), Anti-His (12689, Cell Signaling Technology), anti-HA (3724, Cell Signaling Technology),

MDA (ab27642, Abcam), 4-HNE (ab48506, Abcam), MG132 (S1748, Beyotime), cycloheximide (HY12320, MedChemExpress), Chloroquine (HY-17589A, MedChemExpress), Onvansertib (HY-15828, MedChemExpress), MLN0905 (HY-15155, MedChemExpress), Recombinant Human PLK1 (ab271716, Abcam).

### Immunoprecipitation (IP) and immunoblotting (IB)

For IB analysis, cell lysis buffer (P0013, Beyotime) supplemented with PMSF (ST506, Beyotime) and phosphatase inhibitor cocktail was used to lyse the cells (P1081, Beyotime). Proteins were isolated using polyacrylamide gel electrophoresis with sodium dodecyl sulfate and then transferred to polyvinylidene fluoride (PVDF) membranes. The membranes were blocked with 5% non-fat milk and the indicated primary antibodies at 4 °C overnight. After being washed three times with TBST buffer, the cells were incubated with the HRP-conjugated secondary antibodies (SA00001-1, SA00001-2; Proteintech) and detected by using chemiluminescence (34580, Thermo Fisher).

For IP analysis, cell lysis buffer (P0013, Beyotime) containing PMSF and phosphatase inhibitor cocktail was used to lyse the cells for IP analysis (P1081, Beyotime). Cell lysates were first precleared with protein A/G agarose, and then were incubated with the indicated primary antibodies at 4 °C overnight. The next day, 40 μl of protein A/G-agarose beads were added and incubated for 4 h at 4 °C. After washing three times, the mixture was resuspended. After boiling and centrifugation to pellet the agarose beads, supernatants were subjected to IB analysis.

### Mass spectrometry analysis of GPX4-interacting proteins

HEK293T cells transfected with Myc-GPX4 were subjected to immunoprecipitation (IP) with an anti-Myc antibody or IgG control. Immune complexes were then incubated with Protein A/G Agarose (P2197, Beyotime). After washing 4 times, the immunoprecipitates were boiled in 1X loading buffer and resolved using SDS-PAGE. MS analysis was then performed by Beijing Genomics institution (Beijing, China).

### Xenotransplantation

Six-week-old female mice obtained from Vital River (Beijing, China) were used for in vivo research. Mice were randomized to each experimental group. T98G or U118 cells (1 × 10^5^) transfected as indicated were injected intracranially into the right striatum of each mouse. Tumor growth was monitored by bioluminescent imaging using the IVIS 200 Spectrum system and quantified by Living Image software. At the end of the experiments, the mice were humanely killed, and each mouse’s brain was harvested, fixed in 4% formaldehyde, and embedded in paraffin.

### Real-time PCR and RNA interference

Total RNA was isolated from the cells and tissues using the the TRIzol LS Reagent (Invitrogen). The RNA concentration was then measured using a single instrument. A PrimeScript RT reagent kit was used to reverse-transcribe the recovered RNA into cDNA (RR047A, Takara). Then, using the cDNA as the template and a 20 μL reaction volume, quantitative real-time PCR was performed using an Applied Biosystems 7500 Fast Real-Time PCR System. The 2-ΔΔCT technique was used to quantify relative gene expression, which was then normalized to that of the reference control GAPDH. The primers were as follows: GAPDH forward, 5′-GTGGACATCCGCAAAGACC-3′; GAPDH reverse, 5′-CCTAGAAGCA TTTGCGGTG-3′. GPX4 forward, 5′- CGGAATTCATGAGCCTCGGCCGCCTTTG-3′; GPX4 reverse, 5′- CCGCTCGAGGAAATAGTGGGGCAGGTCCT-3′.

SiRNAs were transfected by using Lipofectamine 3000 (L3000075, Invitrogen™). PLK1 siRNAs were obtained from Cell Signaling Technology (siRNA#1: 6292 S) and Sigma-Aldrich (siRNA#2: EHU051011).

### Plasmids, lentiviral vectors, shRNA, and transfection

TRIM26, GPX4, and PLK1 coding regions were tagged with Flag, Myc and His, respectively, and cloned into the pCDH-CMV-MCS-EF1α-Puro vector. Plasmids encoding HA-labeled ubiquitin and its mutants (#18712, #121151, #121152, #22902, #17607, #17606, #17605, and #17604) were acquired from Addgene. A point mutation in TRIM26 and PLK1 was performed using the QuikChange Mutagenesis Kit (Agilent Technologies). DNA sequencing was performed to verify these constructs. Lentiviral shRNA plasmids targeting TRIM26 were obtained from Dharmacon (Shanghai, China). The sequences were as follows: shRNA#1, 5′-GATGGATATGACGACTGGGAA-3′; shRNA#2, 5′-GCTGCTGAGAGACTTGGAATA-3′. Transfection of the plasmids and shRNA was performed using Lipo3000 (Invitrogen).

### In vivo and in vitro ubiquitination assay

We used denaturing immunoprecipitation (d-IP) to carry out an in vivo GPX4 ubiquitination experiment. After treatment with 20 μM MG132 for 6 h, cells were collected using denatured lysis solution (1.5% β-mercaptoethanol, 62.5 mM Tris-HCl pH 6.8, 10% glycerol, 2% SDS) and boiled for 10 min. After centrifugation for 5 min, immunoprecipitation (IP) and immunoblotting (IB) were performed with indicated the antibodies.

For the in vitro ubiquitin assay of GPX4, purified GPX4 was incubated with Flag-TRIM26, UBE1 (SRP0400, Sigma-Aldrich), UBE2D3 (ab269098, Acbam) and the indicated His-tagged-ubiquitin mutants at 37 °C for 45 min in 40 μl reaction buffer (25 mM Tris pH 7.4, 5 mM MgCl_2_ and 2 mM DTT). The supernatant was then collected by centrifugation for 5 min and then boiled at 95 °C for 5 min after the addition of SDS loading buffer. Ni-NTA beads were used to pull down ubiquitinated GPX4. The eluted proteins were analyzed by IB.

### Cell survival assay

To monitor cell survival, CCK-8, colony formation and trypan blue assays were performed. Each group underwent at least three repeats.

For the CCK8-assay, approximately 2000 tumor cells were plated in each well of a 96-well plate. The foregoing conditions were followed by the addition of 10 µL of CCK-8 reagent (C0037, Beyotime) to each well. Using a microplate reader, the absorbance at 450 nm was calculated.

Cell proliferation was also detected by colony formation assay. The procedure involved cultivating cells (300/well) on 6-well plates for 14 days. Cells were fixed with 4% formaldehyde for 10 min, and then were stained with crystal violet (G1014, Servicebio) at room temperature for 30 min before calculation.

Cell viability was determined by Trypan Blue assay. After the indicated treatment, the cells were incubated with trypan blue solution (ST798, Beyotime) for 5 min, and the number of living cells (Trypan Blue negative) from five random fields was counted using ImageJ software (NIH, Bethesda, MD).

### Protein purification and GST pull-down

For GST-GPX4 purification, the GST-GPX4 bacterial expression plasmid pGEX-4T-1 was expressed in Escherichia coli BL21. Following centrifugation, the cell lysates were incubated with glutathione beads (G0924, Sigma) overnight at 4 °C and then GST-GPX4 was purified according to the manufacturer’s protocol. Flag-TRIM26 and its mutant were transfected into HEK293T cells, and then Flag-tagged proteins were purified using anti-Flag magnetic beads (P2115, Beyotime). The recombinant proteins were examined using SDS-PAGE and stored at −80 °C.

For the GST pull-down assay, Flag-TRIM26 or its mutants were incubated with the purified GST or GST-GPX4 bound to glutathione-Sepharose 4B beads (17-0756-01, GE Healthcare) for 12 h at 4 °C in the binding buffer. After washing three times with binding buffer, the mixture was subjected to IB analysis. GST-fusion proteins and GST were assessed by Coomassie Blue staining.

### Duo-link proximity ligation assay (PLA)

For the PLA assay, 5 × 10^3^ cells were seeded into a confocal dish. Following fixation with 4% paraformaldehyde, the cells were permeabilized, blocked and probed with the indicated antibodies. Afterwards, the Duolink® In Situ Red Mouse/Rabbit kit (DUO92101, Sigma Aldrich) was used to detect the PLA foci according to the manufacturer’s protocol.

### Immunofluorescent (IF) staining

For IF staining, cells were seeded in confocal dishes at the appropriate density. After fixation with 4% paraformaldehyde, permeabilization with 0.5% Triton X-100, and blocking with regular goat serum, the cells were incubated with the indicated primary antibodies at 4 °C overnight. The cells were washed three times with PBS and then incubated with Alexa Flour 488- or 594-labeled secondary antibodies (A11001, A11008, A11037, and A11032; Thermo Fisher). Images were then captured with a confocal microscope and analyzed using LAS X software.

### In vitro kinase assay

Purified Flag-TRIM26 and its Flag-tagged mutant protein were incubated with 10 ng of recombinant PLK1 in the reaction buffer containing 2 µCi[γ32P] ATP per reaction. The in vitro kinase reaction was conducted at 30 °C for 30 min, and the reaction was stopped by adding SDS loading buffer. The resultant product was then subjected to SDS-polyacrylamide gel electrophoresis and autoradiography.

### Lipid peroxidation and ROS analysis

Lipid peroxidation was determined by the detection of intracellular MDA and 4-HNE levels using the Lipid Peroxidation MDA Assay Kit (ab118970, Abcam) and the Lipid Peroxidation 4-HNE Assay Kit (ab238538, Abcam).

The Reactive Oxygen Species (ROS) Assay Kit (S0033M, Beyotime) was used to measure the level of ROS. FACS analysis was performed using a flow cytometry (FACSCalibur, BD Biosciences).

### Statistical analysis

Analyses were carried out using GraphPad Prism software (Version 7.0). Data are represented as the mean ± SD. Two-tailed Student’s t-tests and one-way ANOVAs were utilized in the data analysis for the article. A Kaplan-Meier model was used to conduct survival analysis. A *P*-value less than 0.05 was considered to be statistically significant.

## Result

### TRIM26 maintains GPX4 protein stability via direct binding

**Fig. 1:**
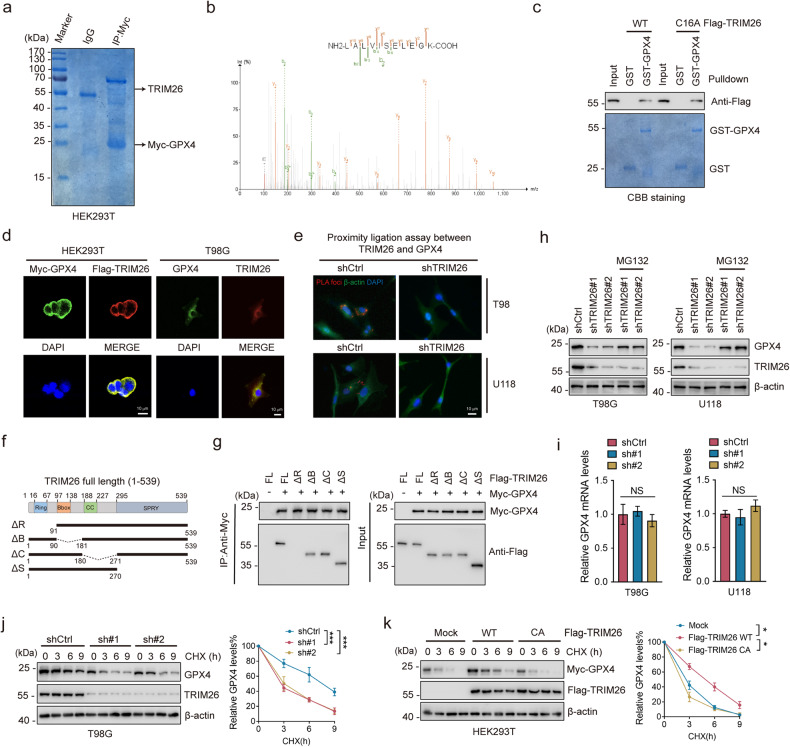
TRIM26 maintains GPX4 protein stability via direct binding. **a** SDS-PAGE and Coomassie brilliant blue (CBB) staining of Myc-immunoprecipitated proteins from HEK293T cells stably overexpressing Myc-GPX4. Arrows indicate the proteins of interest. **b** Peptides of TRIM26 identified by mass spectrometry (MS) analysis. **c** GST-pulldown assay between TRIM26 and GPX4. Bacterially produced GST control and GST-GPX4 were visualized by CBB staining. Flag-TRIM26 WT or Flag-TRIM26 CA were visualized by gel electrophoresis. **d** Immunofluorescence (IF) staining of GPX4 (green), TRIM26 (red), and DAPI (blue) in HEK293T and T98G cells. Scale bars, 10 μm. **e** Images of PLA for endogenous GPX4 and TRIM26 in T98G and U118 cells transfected as indicated. **f** Sketches of full-length (FL) TRIM26 and four TRIM26 deletion mutants. **g** Flag-TRIM26 FL or its deletion mutants were co-transfected with the Myc-GPX4 into HEK293T cells. Cell lysates were subjected to immunoprecipitation (IP) using an anti-Myc antibody and then analyzed by immunoblotting (IB). **h** T98G and U118 cells were transfected with TRIM26 shRNA. After treatment with or without MG132 (20 µM) for 6 h, cell lysates were subjected to IB. **i** Quantitative real-time PCR (qPCR) for mRNA levels of GPX4 in T98G and U118 cells transfected with TRIM26 shRNAs or not. **j** T98G cells transfected with TRIM26 shRNAs were incubated with cycloheximide (CHX) for indicated times, then cell lysates were subjected to IB. Quantification of GPX4 levels relative to β-actin is shown. **k** Flag-TRIM26 WT or Flag-TRIM26 CA mutant was co-transfected with the Myc-GPX4 into HEK293T cells. After incubation with CHX for indicated times, cells were collected and subjected to IB. Quantification of GPX4 levels relative to β-actin is shown. Data are represented as the mean ± SD (*n* = 3). One-way ANOVA with Dunnett’s post-test (**i**); student’s t-test (**j**, **k**). *P < 0.05, **P < 0.01, ***P < 0.001, NS: non-significance.

To interrogate the underlying regulatory modes of GPX4, we performed a mass spectrometry (MS) analysis to identify novel GPX4-interacting proteins by using HEK293T cells stably expressing Myc-GPX4 (Fig. 1a). As the top candidate, TRIM26, belonging to the tripartite motif (TRIM) family proteins and participating in the regulation of its substrate at the post transcriptional level, caught our attention (Fig. 1b and Supplementary Table S1). We next confirmed the endogenous and exogenous interaction between TRIM26 and GPX4 (Supplementary Fig. S1a–c). The GST pull-down assay further determined the direct binding between TRIM26 and GPX4 which was independent of the catalytic activity of TRIM26 (Fig. 1c). Moreover, confocal analysis showed colocalization of TRIM26 and GPX4 in the cytoplasm of HEK293T and T98G cells (Fig. 1d). Consistently, the proximity ligation assay (PLA) demonstrated that the interaction between these proteins mainly occurs in the cytoplasm (Fig. 1e). To determine the structural requirements of the TRIM26-GPX4 interaction, we generated a panel of TRIM26 deletion mutants (ΔR, ΔB, ΔC and ΔS) (Fig. 1f). We found that TRIM26 ΔB, TRIM26 ΔC and TRIM26 ΔS were able to bind GPX4, whereas TRIM26 ΔR was not, indicating that the Ring domain of TRIM26 is required for its binding to GPX4 (Fig. 1g).

TRIM26 is implicated in various biological processes by catalyzing ubiquitin-proteasome-mediated degradation of its substrate [23]. Thus, we wonder that whether TRIM26 plays a role in GPX4 stability. Interestingly, cells with TRIM26 knockdown exhibited a decreased level of GPX4 protein abundance and this effect was reversed by the addition of the proteasome inhibitor MG132 (Fig. 1h), but not by the lysosomal inhibitor chloroquine (Supplementary Fig. S1d). In contrast, forced expresion of TRIM26 enhanced GPX4 protein levels in a dose-dependent manner (Supplementary Fig. S1e). Notably, neither TRIM26 knockdown nor TRIM26 overexpression altered the mRNA levels of GPX4 (Fig. 1i and Supplementary Fig. S1f), indicating that TRIM26 regulates GPX4 expression at the post-transcriptional level. To further determine the effect of TRIM26 on GPX4 stability, we conducted cycloheximide (CHX) chase assay. Depletion of TRIM26 markedly promoted the degradation of GPX4 (Fig. 1j and Supplementary Fig. 1g), whereas overexpression of TRIM26 WT, but not its catalytically inactive mutant TRIM26 C16A prolonged the half-life of GPX4 (Fig. 1k). Similar results were further verified in A172 and LN229 cells, in which TRIM26 WT, but not TRIM26 C16A or TRIM26 ΔR prolonged the half-life of GPX4 (Supplementary Fig. 1h, i). Together, these data suggest that TRIM26 is a GPX4-interacting protein responsible for the regulation of GPX4 proteostasis.

### TRIM26 induces the K48-K63-linked polyubiquitination transition of GPX4

**Fig. 2:**
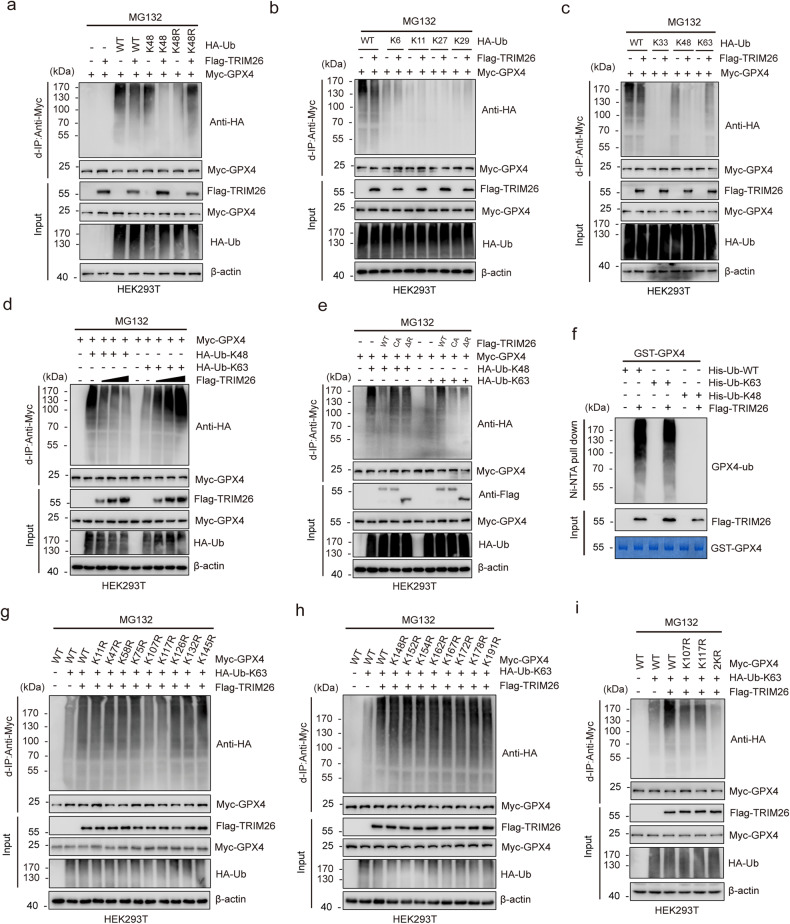
TRIM26 induces the K48-K63-linked polyubiquitination transition of GPX4. **a** HEK293T cells were transfected with Myc-GPX4 and the indicated ubiquitin mutant together with Flag-TRIM26 or not and then treated with MG132 (20 µM) for 6 h. Cell lysates were subjected to d-IP and IB with the indicated antibodies. **b**, **c** HEK293T cells transfected with the indicated plasmids were subjected to denaturing-IP (d-IP) and IB with the indicated antibodies. **d** HEK293T cells were transfected with Myc-GPX4 and indicated HA-Ub together with increasing amounts of Flag-TRIM26 and then treated with MG132 (20 µM) for 6 h. Cell lysates were subjected to d-IP and IB with the indicated antibodies. **e** HEK293T cells were transfected with Myc-GPX4 and indicated HA-Ub together with Flag-TRIM26 WT, Flag-TRIM26 CA or Flag-TRIM26 ΔR and then treated with MG132 (20 µM) for 6 h. Cell lysates were subjected to d-IP and IB with the indicated antibodies. **f** In vitro ubiquitination assay using the indicated purified proteins in the presence of E1, E2, and ATP. The reaction mixtures were incubated with Ni-NTA beads and then subjected to IB with an anti-GPX4 antibody. **g**–**i** HEK293T cells were transfected with Flag-TRIM26 and HA-Ub-K63 together with Myc-GPX4 WT or its mutants and then treated with MG132 (20 µM) for 6 h. Cell lysates were subjected to d-IP and IB with the indicated antibodies.

Protein stability is mainly controlled by the ubiquitination system. As TRIM26 is known to assemble several different types of ubiquitin chains [12, 14, 23], we wondered whether TRIM26 plays a role in GPX4 ubiquitination. To test this, we overexpressed Myc-GPX4 and Flag-TRIM26 with HA-Ub WT, HA-Ub K48 (with other K residues mutated into R except K48), or HA-Ub K48R (with K48 residues mutated into R) in HEK293T cells and conducted denaturing-IP (d-IP) assays. The results showed that cells overexpressing TRIM26 exhibited a lower level of GPX4 K48-linked polyubiquitination and a higher level of GPX4 non-K48-linked polyubiquitination than cells expressing the vector control (Fig. 2a). Polyubiquitination can occur through seven different Lys resides on ubiquitin (K6, K11, K27, K29, K33, K48 and K63) [19, 24]. We thus used a panel of ubiquitin mutants in which only one Lys residue was retained, and we found that TRIM26 enhanced K63-linked polyubiquitination and decreased K48-linked polyubiquitination of GPX4 (Fig. 2b, c). This effect of TRIM26 was dose-dependent (Fig. 2d). In contrast, TRIM26 ΔR or TRIM26 C16A failed to support this finding (Fig. 2e). An in vitro ubiquitination assay further demonstrated that TRIM26 targeted GPX4 for K63-linked ubiquitination (Fig. 2f). Consistent with our findings, tumors cells with TRIM26 knockdown showed higher levels of K48-linked polyubiquitination and lower levels of K63-linked polyubiquitination of GPX4 (Supplementary Fig. S2a–d).

The human GPX4 protein contains 17 Lys residues. To further determine the ubiquitination site(s) of GPX4, we mutagenized every Lys to Arg and co-transfected each GPX4 mutant with HA-Ub K63 and Flag-TRIM26 into HEK293T cells. Two mutations (K107R and K117R) displayed a reduction in TRIP26-mediated ubiquitination (Fig. 2g, h). The combined mutation, K107R/K117R (denoted as 2KR), exhibited a further decrease in ubiquitination (Fig. 2i). Collectively, these results clearly suggest that TRIM26 mediates the K63-linked ubiquitination of GPX4 at K107 and K117.

### TRIM26 suppresses ferroptosis in glioma cells

To determine the role of TRIM26 in glioma, we evaluated the expression of TRIM26 using RNA sequencing datasets from GEPIA 2 (http://gepia2.cancer-pku.cn/#index). We found that the mRNA levels of TRIM26 in glioblastoma (GBM) and low-grade gliomas (LGG) were higher than those in normal brain tissues (NB) (Supplementary Fig. S3a). Immunoblot assays showed that the protein level of TRIM26 was almost undetectable in normal human astrocytes (NHAs) and markedly higher in a panel of GBM cell lines compared to that in two LGG cell lines (Sw1783 and Hs683) (Supplementary Fig. S3b). Consistently, immunohistochemical (IHC) assays determined the positive association between TRIM26 expression and glioma grade (Supplementary Fig. S3c). These results suggest an oncogenic role of TRIM26 in glioma.

**Fig. 3:**
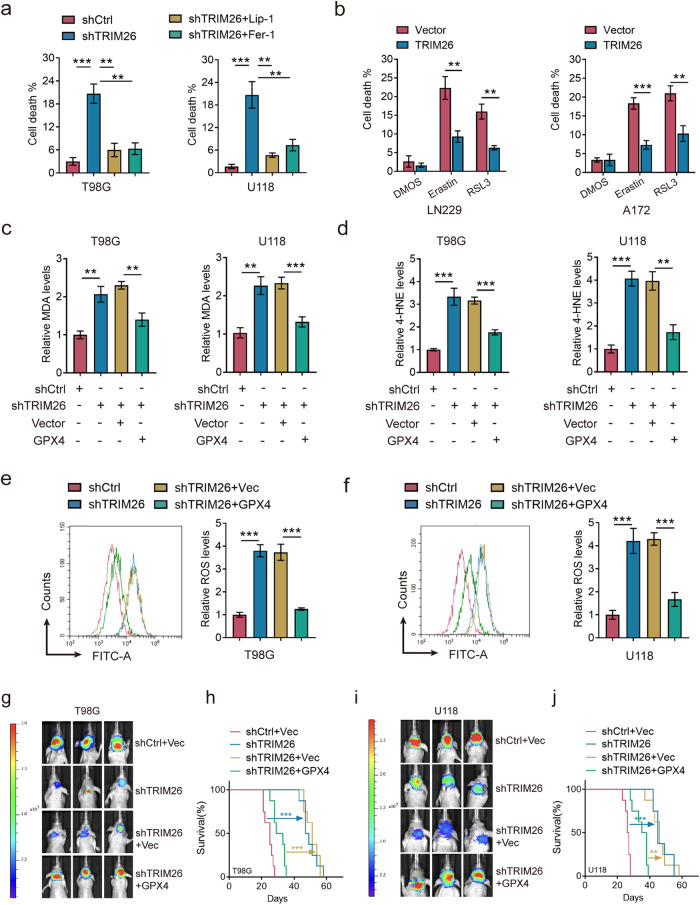
TRIM26 suppresses ferroptosis in glioma cells. **a** Trypan blue staining assays using shCtrl or shTRIM26 T98 and U118 cells treated with ferrostatin-1(100 nM) or liproxstatin-1 (50 nM). Data are represented as mean ± SD (*n* = 5); student’s t test; **P < 0.01, ***P < 0.001. **b** Trypan blue staining assays using LN229 and A172 cells expressing vector control or TRIM26 treated with Erastin (20 µM) or RSL3 (5 µM). Data are represented as mean ± SD (*n* = 5); student t test; **P < 0.01, ***P < 0.001. **c**–**f** Relative MDA (**c**), 4-HNE (**d**) and ROS levels (**e**, **f**) in T98G and U118 cells transfected as indicated. Data are represented as the mean ± SD (*n* = 3); student’s t-test; **P < 0.01, ***P < 0.001. **g**–**j** Luciferase expressing T98G (**g**) and U118 (**i**) cells were transduced with indicated plasmids and then injected **i**nto mice brains. Representative bioluminescent live (BLI) images of three mice are shown. Kaplan-Meier survival curves of mice intracranially injected with T98G (**h**) and U118 (**j**) cells with the indicated modifications (*n* = 8); log-rank (Mantel-Cox) test, **P < 0.01, ***P < 0.001.

Given the catalytic effect of TRIM26 on GPX4, we were motivated to investigate its role in ferroptosis. TRIM26 knockdown substantially impaired the survival of T98G and U118 cells, while this effect was largely reversed by treatment with two ferroptosis inhibitors, ferrostatin-1 and liproxstatin-1(Fig. 3a). In contrast, cell death induced by erastin and RSL3 (two ferroptosis inducers) was rescued by TRIM26 overexpression (Fig. 3b). Moreover, elevated cellular MDA, 4-HNE and ROS levels were observed in TRIM26-knockdown tumor cells, which was reversed by the re-expression of GPX4. (Fig. 3c–f). In contrast, similar to overexpression of GPX4, overexpression of TRIM26 reduced the cellular levels of MDA, 4-HNE and ROS (Fig. S3d–q).

In vivo, knockdown of TRIM26 inhibited, while GPX4 re-expression restored, GBM intracranial tumor growth, which in turn altered the overall survival of tumor-bearing mice (Fig. 3g–j). Taken together, these data indicate that TRIM26 promotes tumorigenesis of glioma by suppressing ferroptosis via GPX4.

### PLK1 phosphorylates TRIM26 at S127

**Fig. 4:**
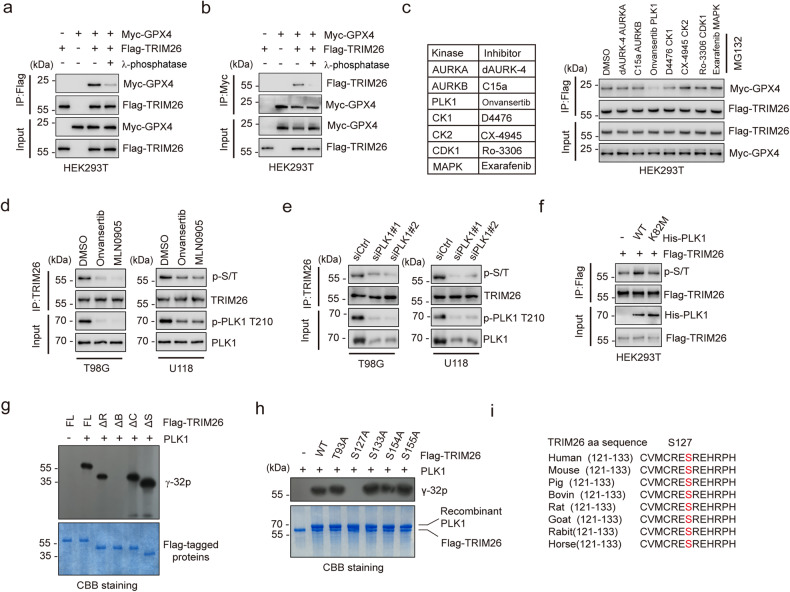
PLK1 phosphorylates TRIM26 at S127. **a**, **b** IB analysis of cell lysates and anti-Flag (**a**) or anti-Myc (**b**) immunoprecipitates derived from HEK293T cells treated as indicated. **c** HEK293T cells transfected with Flag-TRIM26 and Myc-GPX4 were incubated with the indicated inhibitors and then subjected to IP and IB with the indicated antibodies. **d** T98G and U118 cells treated with onvansertib (2 nM) or MLN0905 (2 nM) were subjected to IP and IB with the indicated antibodies. **e** T98G and U118 cells treated with control siRNA or PLK1 siRNAs were subjected to IP and IB with the indicated antibodies. **f** HEK293T cells transfected with Flag-TRIM26 and His-PLK1 WT or His-PLK1 K82M were subjected to IP and IB with the indicated antibodies. **g**, **h** The Flag-TRIM26 FL or the indicated mutants were purified from transfected HEK293T cells and were used for the in vitro PLK1 assay as a substrate. Phosphorylation was determined by ^32^P-autoradiogram and purified proteins were determined by CBB. **i** Sequence alignment around S127 in TRIM26.

Multiple TRIM family proteins have been reported to be phosphorylated which is required for the recognition of their substrates [25, 26], indicating that phosphorylation state of TRIM family proteins is required for their enzymatic activity. To examine whether phosphorylation is required for TRIM26 activity, we treated immunoprecipitated TRIM26 and GPX4 with λ-phosphatase to dephosphorylate these proteins and found that the interaction between TRIM26 and GPX4 was severely reduced (Fig. 4a, b). This prompted us to investigate the putative kinase responsible for the TRIM26-GPX4 interaction. To this end, we treated HEK293T cells with a panel of inhibitors targeting the kinases identified by MS above (Supplementary Table S1). As shown in Fig. 4c, onvansertib, a highly selective inhibitor of PLK1, markedly reduced the interaction between TRIM26 and GPX4. To determine whether PLK1 has an effect on TRIM26 or GPX4, we monitored the phosphorylation levels of TRIM26 and GPX4 using an antibody against pan phospho-serine/threonine (p-S/T). In fact, PLK1 inhibition with either onvansertib or MLN0905 severely diminished phosphorylation levels of TRIM26, but not that of GPX4 (Fig. 4d and Supplementary Fig. S4a, b). Consistently, knockdown of PLK1 decreased the p-S/T of TRIM26 (Fig. 4e), whereas overexpression of PLK1 WT, but not the kinase-dead K82M mutant, enhanced the p-S/T of TRIM26 (Fig. 4f). Moreover, we found that TRIM26 phosphorylation levels were correlated with PLK1 activity in a panel of glioma cell lines (Supplementary Fig. S4c) and confirmed endogenous binding between PLK1 and TRIM26 (Supplementary Fig. S4d), suggesting that PLK1 is a key kinase that targets TRIM26.

Next, we purified TRIM26 deletion mutants and performed an in vitro kinase assay to determine the phosphorylation site(s) of TRIM26. We found that PLK1 phosphorylated the Bbox domain (aa 91-180) of TRIM26 (Fig. 4g). We then mutagenized each serine/threonine (S/T) to alanine (A) and found that phosphorylation was undetectable in the TRIM26 S127A mutant but was present in other mutants of the TRIM26 protein (Fig. 4h). Consistently, forced expression of PLK1 increased the p-S/T of TRIM26 WT, but not that of TRIM26 S127A (Supplementary Fig. S4e). In addition, the aa sequence of TRIM26 surrounding S127 is conserved among multiple species (Fig. 4i). These data strongly suggest that PLK1 specifically phosphorylates TRIM26 at Ser 127.

### PLK1-mediated TRIM26 phosphorylation increases GPX4 stability

**Fig. 5:**
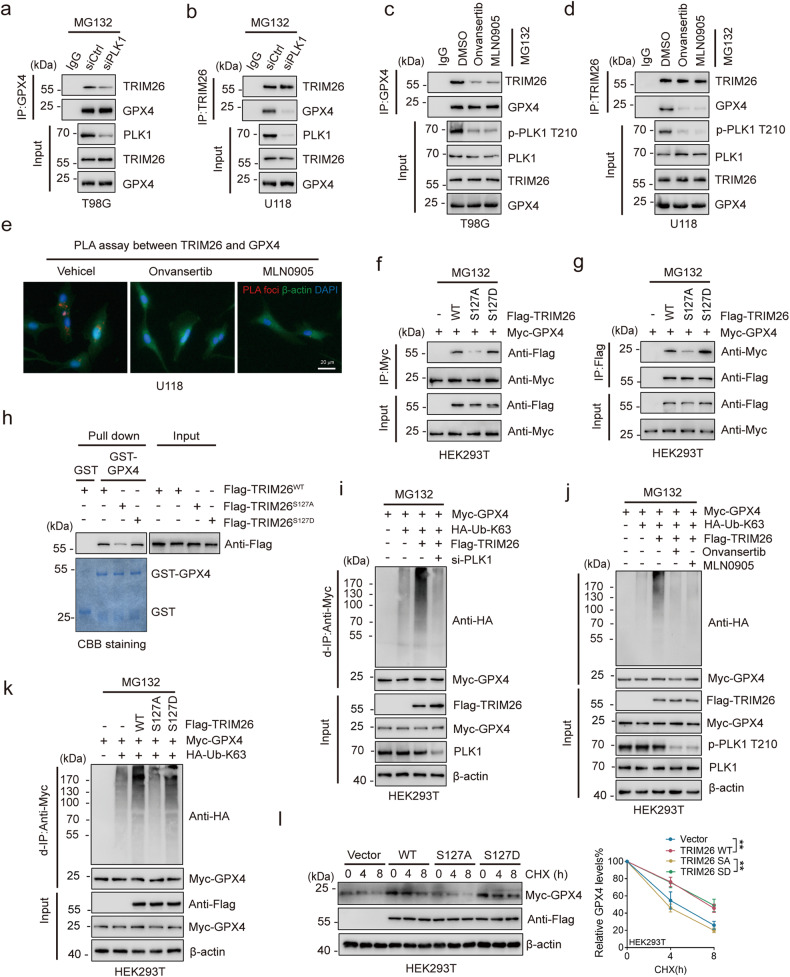
PLK1-mediated TRIM26 phosphorylation increases GPX4 stability. **a**, **b** T98G (**a**) and U118 (**b**) cells transfected with control siRNA or PLK1 siRNA were subjected to IP and IB with the indicated antibodies. **c**, **d** T98G (**c**) and U118 (**d**) cells treated as indicated were subjected to IP and IB with the indicated antibodies. **e** PLA assay between TRIM26 and GPX4 in U118 cells treated with onvansertib (2 nM) or MLN0905 (2 nM). Scale bar: 20 μm. **f**, **g** Flag-TRIM26 WT, Flag-TRIM26 S127A or Flag-TRIM26 S127D was co-transfected with Myc-GPX4 into HEK293T cells. After treated with MG132 (20 µM) for 6 h. Cell lysates were subjected to d-IP and IB with the indicated antibodies. **h** Purified Flag-TRIM26 WT, Flag-TRIM26 S127A or Flag-TRIM26 S127D was incubated with GST or GST-GPX4 conjugated to beads. Pull-down samples and 5% of the input were analyzed by IB. Purified GST and GST-GPX4 were determined by CBB. **i** HEK293T cells transfected as indicated were subjected to d-IP with anti-Myc antibody and then analyzed by IB. **j** HEK293T cells treated as indicated were subjected to d-IP with anti-Myc antibody and then analyzed by IB. **k** HEK293T cells transfected as indicated were subjected to d-IP with anti-Myc antibody and then analyzed by IB. **l** Flag-TRIM26 WT, Flag-TRIM26 S127A or Flag-TRIM26 S127D was co-transfected with Myc-GPX4 into HEK293T cells. After treated with CHX for the indicated times, then cell lysates were subjected to IB. Quantification of GPX4 levels relative to β-actin is shown; student’s t test; **P < 0.01.

To further investigate the effect of TRIM26 phosphorylation on its binding with GPX4, we knocked down PLK1 in T98G and U118 cells using small interfering RNA (siRNA) and observed a dramatic decrease in the interaction between TRIM26 and GPX4 (Fig. 5a, b). Consistently, the endogenous TRIM26-GPX4 interaction was diminished by PLK1 inhibition with onvansertib and MLN0905 (Fig. 5c, d), which was further validated by a Duo-link assay (Fig. 5e). Moreover, compared with TRIM26 WT and pseudo-phosphorylation mutant TRIM26 S127D, non-phosphorylatable TRIM26 S127A exhibited a weaker interaction with GPX4 (Fig. 5f, g), which was further confirmed by the GST pull-down assay (Fig. 5h).

Consistent with our result above that TRIM26 mediates GPX4 stability, PLK1 depletion or inhibition led to a marked decrease in GPX4 protein levels (Fig. S5a, b). Moreover, the decay rates and K63-linked polyubiquitination of GPX4 decreased upon PLK1 knockdown or PLK1 inhibition (Fig. 5i, j and Supplementary Fig. 5c–f). Accordingly, co-expression of TRIM26 WT or TRIM26 S127D promoted GPX4 K63-linked polyubiquitination and protein stability, while the TRIM26 S127A mutant did not support this effect (Fig. 5k, l). Taken together, these observations reveal that PLK1-mediated phosphorylation of TRIM26 promotes the interaction between TRIM26 and GPX4, leading to GPX4 stabilization.

### S127 phosphorylation is required for TRIM26 function in ferroptosis

**Fig. 6:**
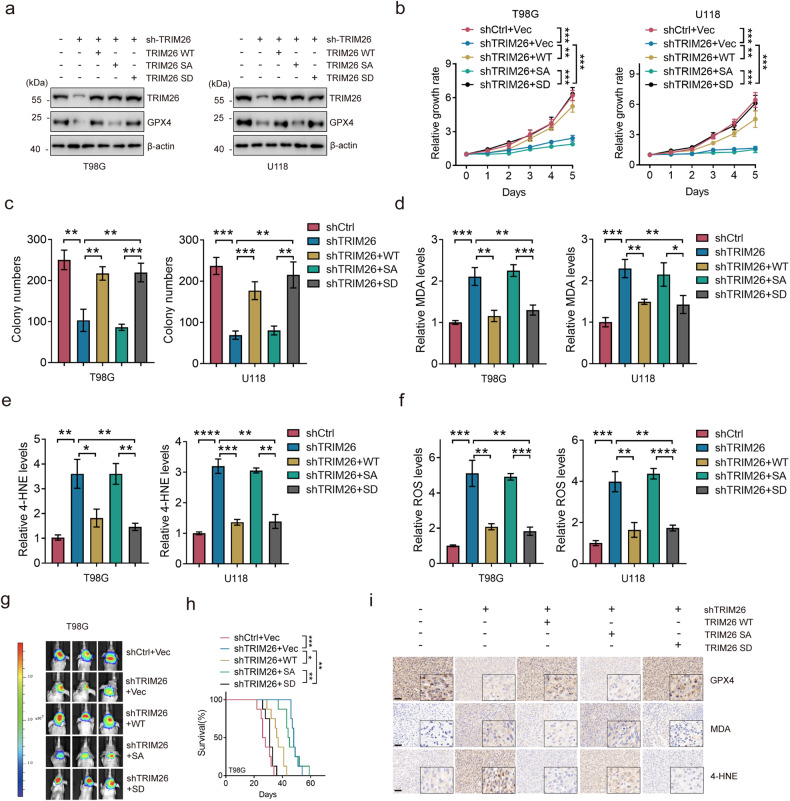
S127 phosphorylation is required for TRIM26 function in ferroptosis. **a** T98 and U118 cells were transfected as indicated, and the TRIM26 and GPX4 expression levels were determined by IB. **b**, **c** Cell proliferation of T98 and U118 cells transfected as indicated using CCK-8 assay (**b**) and colony formation assay (**c**). Data are represented as the mean ± SD (*n* = 3); student’s t-test; **P < 0.01, ***P < 0.001. **d**–**f** Relative MDA (**d**), 4-HNE (**e**), and ROS levels (**f**) in T98G and U118 cells transfected as indicated. Data are represented as the mean ± SD (*n* = 3); student’s t test; *P < 0.05, **P < 0.01, ***P < 0.001. **g** Luciferase expressing T98G cells were transduced with indicated plasmids and then injected into mouse brains. Representative bioluminescent live (BLI) images of three mice were shown. **h** Kaplan-Meier survival curves of mice intracranially injected with T98G cells with the indicated modifications (*n* = 8); log-rank (Mantel-Cox) test, **P < 0.01, ***P < 0.001. **i** Representative IHC images of G*P*X4, MDA and 4-HNE in xenografts derived from T98G cells with the indicated modifications. Scale bars, 100 µm.

To examine the biological significance of TRIM26 phosphorylation, we re-introduced TRIM26 WT, S127A and S127D mutants into TRIM26-knockdown T98G and U11 8 cells (Fig. 6a) and overexpressed TRIM26 WT, S127A and S127D in A172 and LN229 cells in which TRIM26 expression was relatively low (Supplementary Fig. 6a). As expected, the expression of TRIM26 S127D, but not the S127A mutant rescued cell survival in T98G and U118 cells with TRIM26 depletion (Fig. 6b, c). Accordingly, erastin-induced defective proliferation was significantly compromised in cells overexpressing the TRIM26 S127D mutant (Supplementary Fig. S6b, c). Moreover, elevated MDA, 4-HNE and ROS levels were observed in TRIM26-knockdown cells, which were rescued by re-expression of TRIM26 S127D (Fig. 6d–f). Accordingly, forced expression of TRIM26 S127D, but not TRIM26 S127A, conferred resistance to erastin in A172 and LN229 cells (Supplementary Fig. 6d–f). In vivo, the inhibitory effect of TRIM26 depletion on glioma tumorigenicity was rescued by TRIM26 S127D, but not by TRIM26 S127A (Fig. 6g, h). The reduction in GPX4 expression and the elevation of MDA and 4-HNE were also validated in TRIM26-knockdown tumors, which were reversed by re-expression of TRIM26 WT and TRIM26 S127D (Fig. 6i). Taken together, these results indicate that TRIM26 S127 phosphorylation is associated with resistance to ferroptosis in glioma cells.

### Clinical relevance between TRIM26, GPX4 and PLK1

**Fig. 7:**
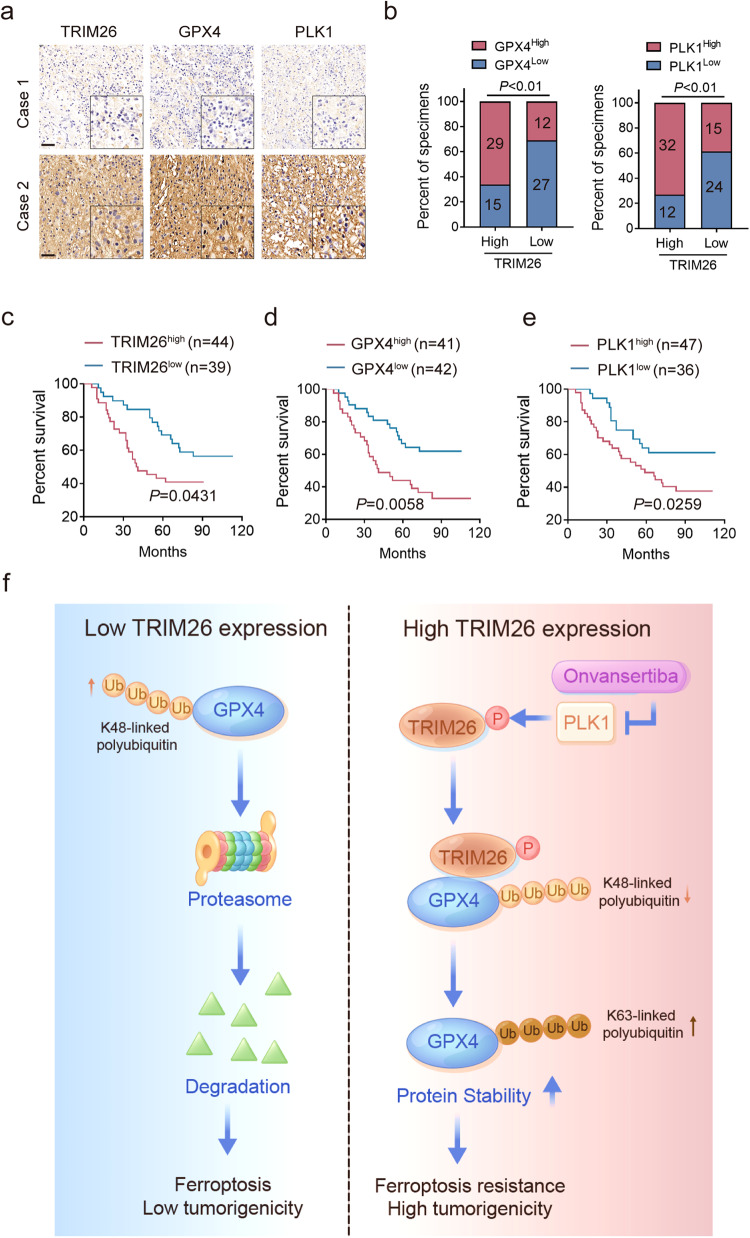
Clinical relevance between TRIM26, GPX4 and PLK1. **a** IHC staining of 83 human glioma samples. Representative images of two specimens are shown. Scale bars, 100 µm. **b** Correlation of TRIM26, GPX4 and PLK1 expression in (**a**); Chi-square test. **c**–**e** Kaplan-Meier curves showing the overall survival of 83 glioma patients divided based on TRIM26 (**c**), GPX4 (**d**) and PLK1 (**e**) expression; log-rank test. **f** The mechanistic scheme of this study.

Finally, we performed an IHC assay on 83 human glioma samples, and observed that TRIM26 expression was positively correlated with GPX4 and PLK1 protein levels (Fig. 7a, b). Moreover, high expression of TRIM26, GPX4 and PLK1 was correlated with poor survival in patients with glioma (Fig. 7c–e).

## Discussion

In this study, we identified TRIM26 E3 ubiquitin ligase as a key regulator of GPX4 proteasomal degradation in glioma cells. Our data revealed that TRIM26 directly interacts with GPX4 through its Ring domain and attaches K63-linked ubiquitin chains on K107 and K117 of GPX4, protecting GPX4 from proteasomal degradation. Moreover, we also demonstrated that PLK1-induced S127 phosphorylation of TRIM26 is essential for TRIM26 to recognize GPX4. The inhibition of this modification impairs GPX4 protein stability, leading to ferroptosis in glioma cells. These data highlight the pivotal role of the PLK1/TRIM26/GPX4 axis in glioma tumorigenesis, suggesting a promising strategy for anti-glioma therapy.

Ferroptosis is a novel type of cell death with distinct properties and functions involved in physical conditions or various diseases and the study of ferroptosis in human cancers is a fast-growing field [27, 28]. GPX4 is identified as the essential regulator of ferroptosis, which converts lipid hydroperoxides into non-toxic lipid alcohols, protecting cells from ferroptosis [29]. Until recently, research show that GPX4 can undergo post-translational modifications (PTMs), such as ubiquitination [30]. It has been reported that the compounds DMOCPTL and bufotalin could directly target GPX4 and induce GPX4 ubiquitination and degradation [31]. Moreover, TRIM46 and TRIM21 were identified as the E3 ligases responsible for GPX4 polyubiquitination [32, 33]. However, the ubiquitination sites and the ubiquitination type of GPX4 remain largely unknown. In this study, we identified TRIM26 as a novel E3 ubiquitin ligase of GPX4 and demonstrated that TRIM26 catalyzes K63-linked polyubiquitination of GPX4 at K107 and K117, which reverses K48-linked polyubiquitination of GPX4 and maintains GPX4 protein stability, providing a novel insight into the PTM of GPX4.

Accumulating evidence suggests that TRIM26, a member of the TRIM family proteins, functions as a pro-oncogenic or tumor-suppressor protein in different cancer types. Wang et al. reported that overexpression of TRIM26 impairs proliferation, epithelial-mesenchymal transition (EMT), and glycolysis in papillary thyroid carcinoma (PTC) cells [34]. Li et al. revealed that TRIM26 prevents the progression of hepatocellular carcinoma (HCC) by promoting ZEB1 degradation [12]. TRIM26 is also considered a pro-oncogenic protein in bladder cancer [35]. Herein, we show that TRIM26 expression is unregulated in glioma, and its overexpression indicates a worse survival outcome. Depletion of TRIM26 could induce ferroptosis and inhibit tumorigenesis of glioma cells, indicating its oncogenic role in glioma.

PLK1, an evolutionarily conserved Ser/Thr protein kinase, is well-known for its function in the cell cycle [36]. PLK1-induced phosphorylation of its substrates governs mitosis, centrosome maturation and spindle assembly [37, 38]. Notably, a growing body of evidence indicates an oncogenic role of PLK1 beyond cell cycle regulation [39]. Overexpression of PLK1 has been identified in multiple cancer types, and is associated with cell proliferation, EMT, apoptosis and chemotherapy resistance [40]. However, whether PLK1 plays a role in ferroptosis remains unknown. In this report, we revealed that PLK1-mediated S127 phosphorylation of TRIM26 is required for the interaction between TRIM26 and GPX4. PLK1 inhibition led to ferroptosis in glioma cells by dephosphorylating TRIM26, further resulting in GPX4 degradation. Hence, the combination of PLK1 inhibitors with ferroptosis inducers could be an attractive tactic against tumors.

In conclusion, we showed that GPX4 undergoes TRIM26-mediated K63-linked polyubiquitination, which antagonizes GPX4 K48-linked polyubiquitination and degradation. Moreover, PLK1-dependent S127 phosphorylation of TRIM26 is essential for the interaction between TRIM26 and GPX4. Disruption of the PLK1-TRIM26-GPX4 axis represses glioma tumorigenesis by promoting ferroptosis, suggesting a potential therapeutic strategy against glioma.

### Reporting summary

Further information on research design is available in the Nature Research Reporting Summary linked to this article.

### Supplementary information

Supplementary Figures and figure legends

Supplementary Table S1

Original Data FileOriginal images of western blot

Reporting Summary

## Data Availability

The data generated in this study are available upon request from the corresponding author.
