# Supplementary material for: The E3 ligase TRIM26 suppresses ferroptosis through catalyzing K63-linked ubiquitination of GPX4 in glioma
Source: Cell Death Dis. 2023 Oct 23;14(10):695. doi: 10.1038/s41419-023-06222-z (PMC10593845; doi:10.1038/s41419-023-06222-z)
Supplement: Supplementary file 1 — Supplementary Figures and figure legends [file 41419_2023_6222_MOESM1_ESM.doc]

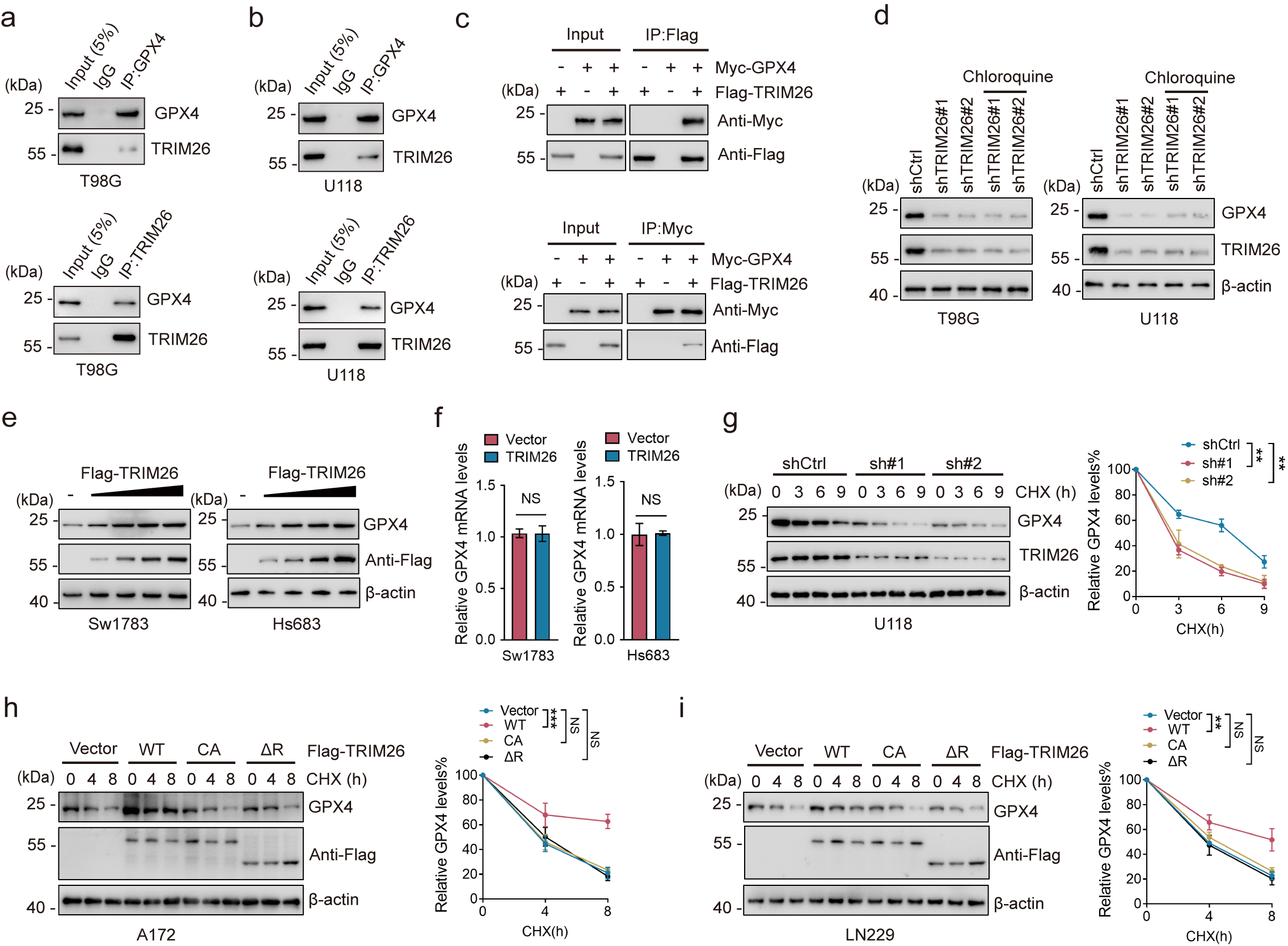


**Fig. S1 TRIM26 maintains GPX4 protein stability via direct binding. a-b** Endogenous interaction between TRIM26 and GPX4. T98G (**a**) and U118 (**b**) cells were subjected to IP using antibodies against TRIM26 or GPX4 and then subjected to IB. Inputs correspond to 5% cell lysates used for immunoprecipitation. **c** HEK293T cells transfected with Flag-TRIM26 and Myc-GPX4 were subjected to IP using antibodies against Flag or Myc and then subjected to IB. **d** T98G and U118 cells were transfected with TRIM26 shRNAs. After treatment with or without chloroquine (25 µM) for 6 h, cell lysates were subjected to IB. **e** Increasing amounts of Flag-TRIM26 were transfected into Sw1783 and Hs683 cells, and cell lysates were subjected to IB. **f** Quantitative real-time PCR for mRNA levels of GPX4 in Sw1783 and Hs683 cells transfected with Flag-TRIM26 or vector control. **g** U118 cells transfected with TRIM26 shRNAs were incubated with CHX for the indicated times, and then cell lysates were subjected to IB. Quantification of GPX4 levels relative to β-actin is shown. **h-i** A172 (**h**) and LN229 (**i**) cells overexpressing TRIM26 WT, TRIM26 CA, or TRIM26 ΔR were incubated with CHX for the indicated times, an then cell lysates were subjected to IB. Quantification of GPX4 levels relative to β-actin is shown. Data are represented as the mean ± SD (n=3); Student’s t-test; **P* < 0.05, ***P* < 0.01, ****P* < 0.001, NS: non-significance.


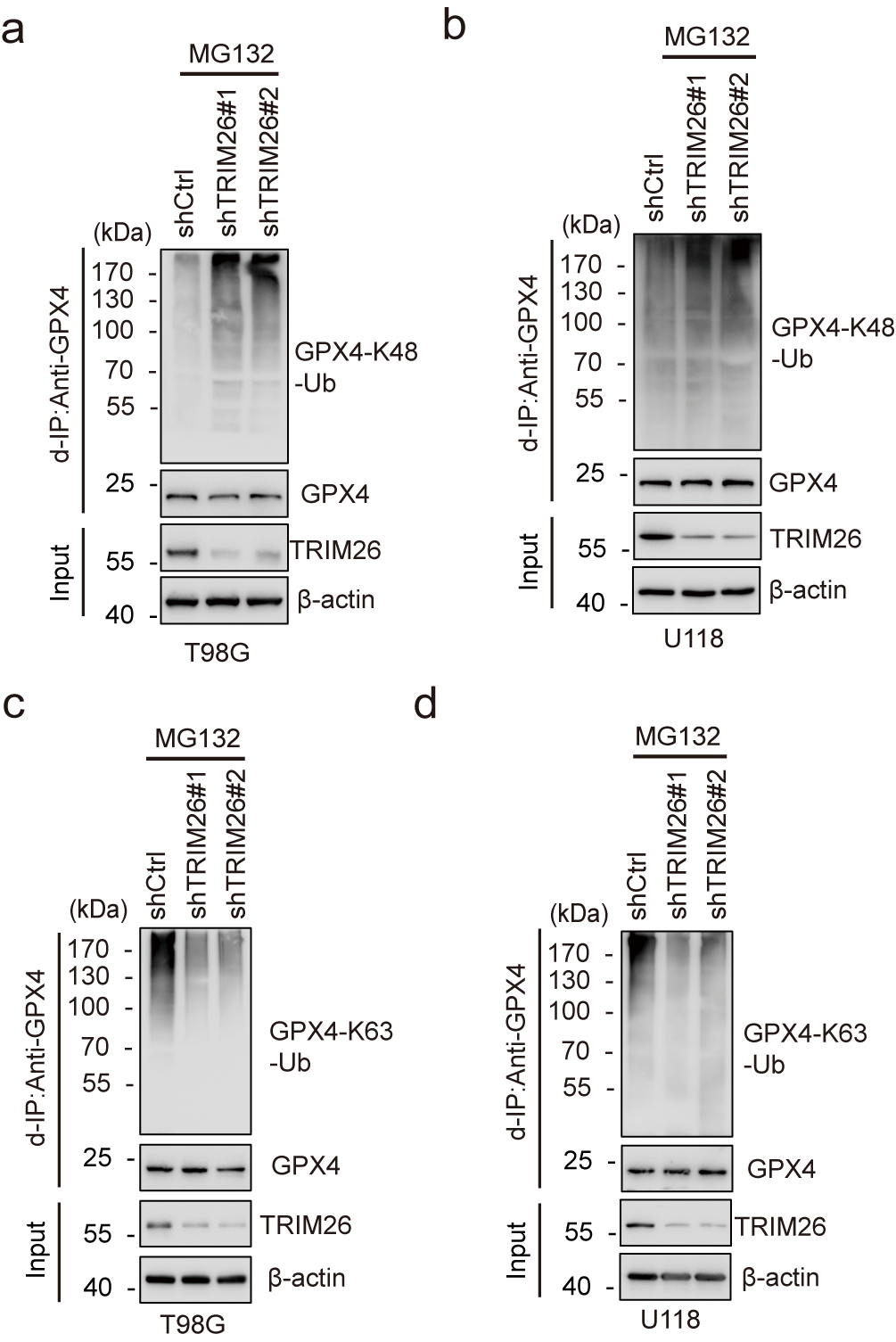


**Fig. S2 TRIM26 induces the K48-K63-linked polyubiquitination transition of GPX4. a-b** T98G (**a**) and U118 (**b**) cells transfected with TRIM26 shRNAs were subjected to d-IP and IB with the indicated antibodies. **c-d** T98G (**c**) and U118 (**d**) cells transfected with TRIM26 shRNAs were subjected to d-IP and IB with the indicated antibodies.


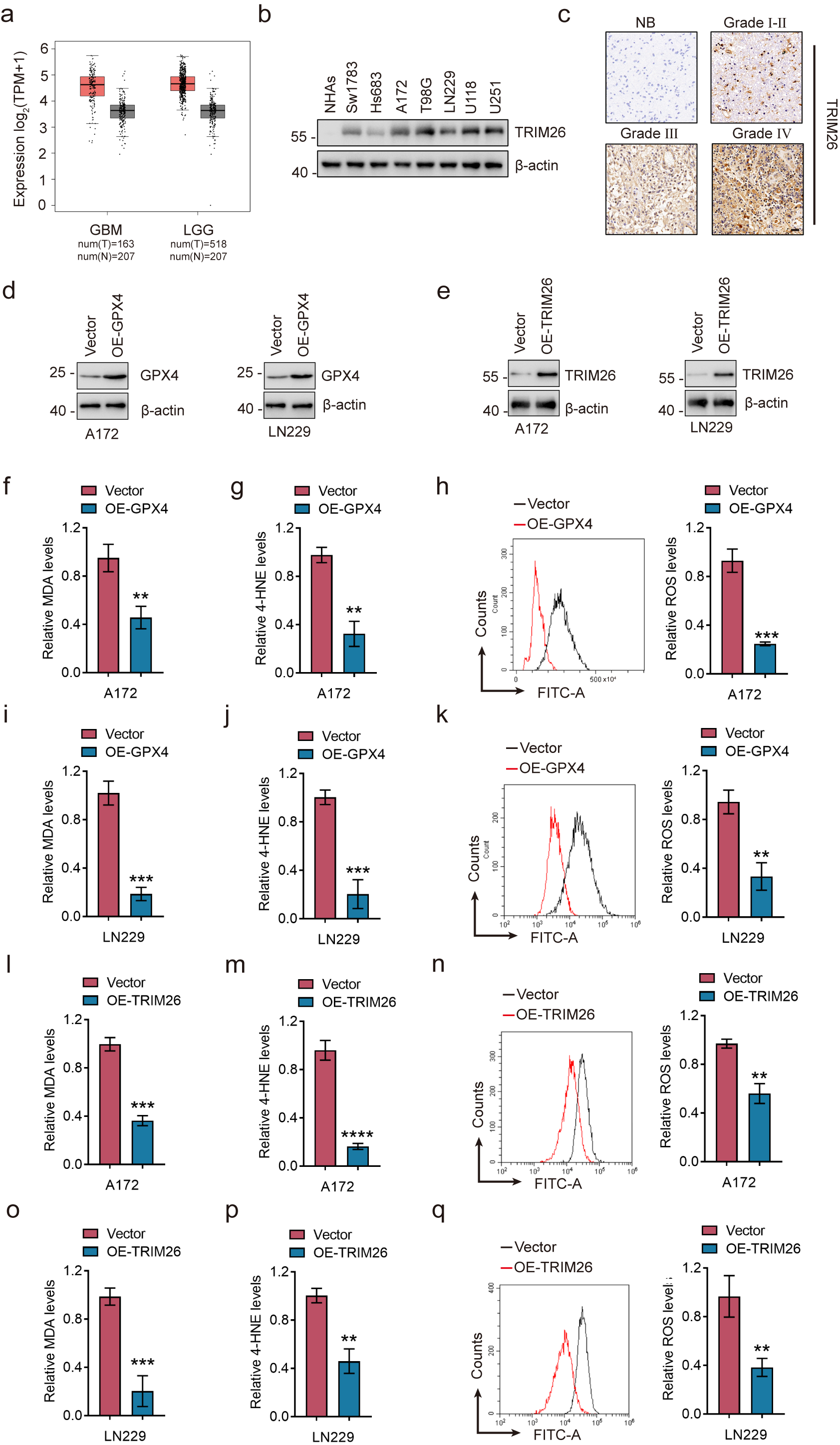


**Fig. S3 TRIM26 suppresses ferroptosis in glioma cells. a** In silico analysis for relative expression levels of TRIM26 in normal brain (NB), low-grade glioma and glioblastoma using the GEPIA 2 website ([http://gepia2.cancer-pku.cn/#index](http://gepia2.cancer-pku.cn/#index)). **b** Relative expression levels of TRIM26 in normal human astrocytes (NHAs) and a panel of glioma cell lines were determined by IB. **c** Representative images of immunohistochemical (IHC) assay for TRIM26 expression in NB and different grades of glioma samples. **d** Western blot assay for GPX4 expression in A172 and LN229 cells. **e** Western blot assay for TRIM26 expression in A172 and LN229 cells. **f-h** Relative MDA (**f**), 4-HNE (**g**), and ROS levels (**h**) in A172 cells overexpressing GPX4 after treatment with erastin (20 µM). **i-k** Relative MDA (**i**), 4-HNE (**j**), and ROS levels (**k**) in LN229 cells overexpressing GPX4 after treatment with erastin (20 µM). **l-n** Relative MDA (**l**), 4-HNE (**m**), and ROS levels (**n**) in A172 cells overexpressing TRIM26 after treatment with erastin (20 µM). **o-q** Relative MDA (**o**), 4-HNE (**p**), and ROS levels (**q**) in A172 cells overexpressing TRIM26 after treatment with erastin (20 µM). Data are represented as the mean ± SD (n=3); student’s t test; ***P* < 0.01, ****P* < 0.001, *****P* < 0.0001.


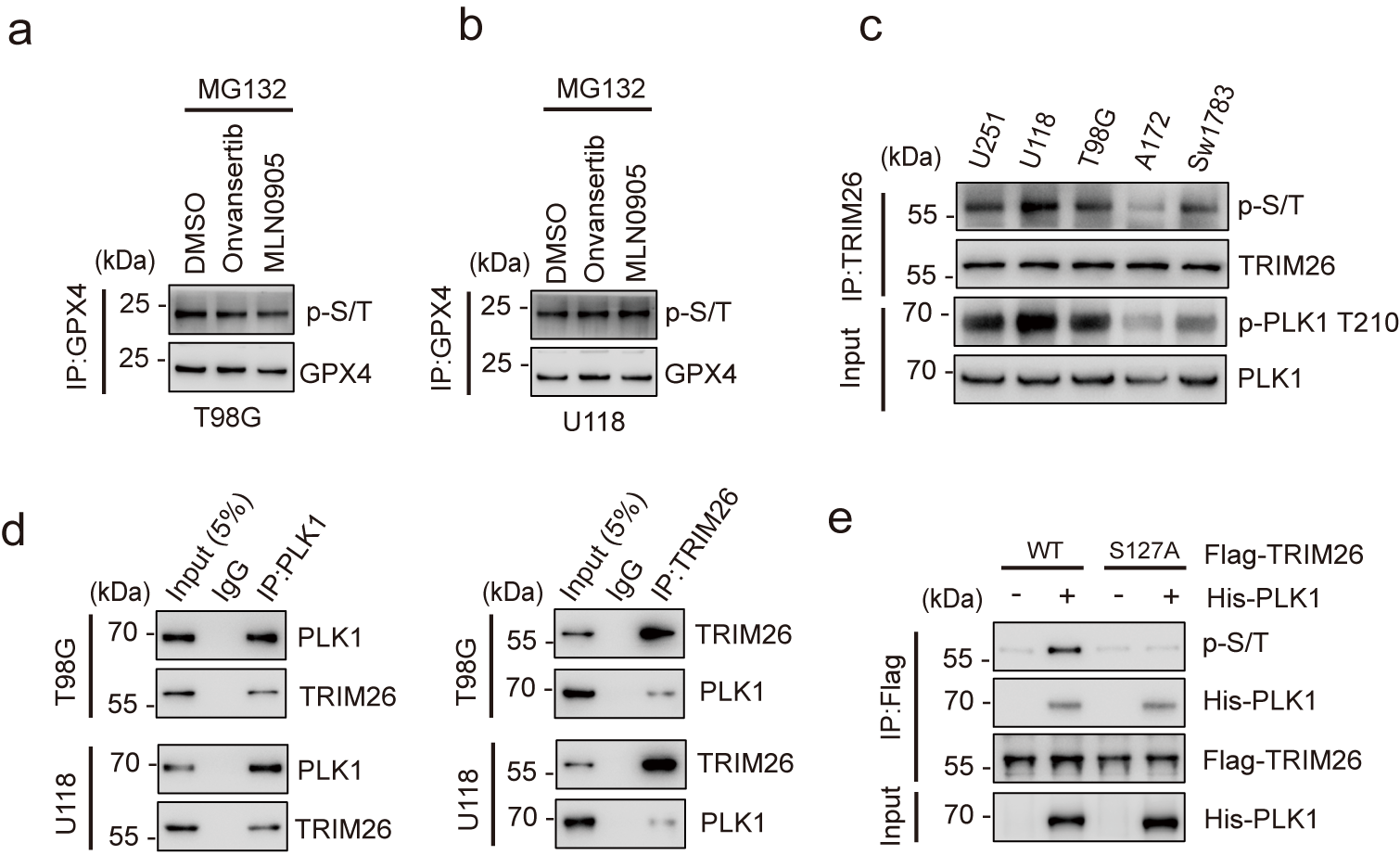


**Fig. S4 PLK1 phosphorylates TRIM26 at S127. a-b** T98G and U118 cells treated as indicated were subjected to IP with anti-GPX4 antibody and IB with the indicated antibodies. **c** IB analysis of cell lysates and anti-TRIM26 immunoprecipitates derived from a panel of glioma cell lines with indicated antibodies. **d** Endogenous interaction betweenTRIM26 and PLK1. T98G and U118 cells were subjected to IP using antibodies against TRIM26 or PLK1, and then subjected to IB. Inputs correspond to 5% cell lysates used for immunoprecipitation. **e** Flag-TRIM26 WT or Flag-TRIM26 S127A were co-transfected with His-PLK1 into HEK293T cells. The cell lysates were subjected to IP with anti-Flag antibody and IB with the indicated antibodies.


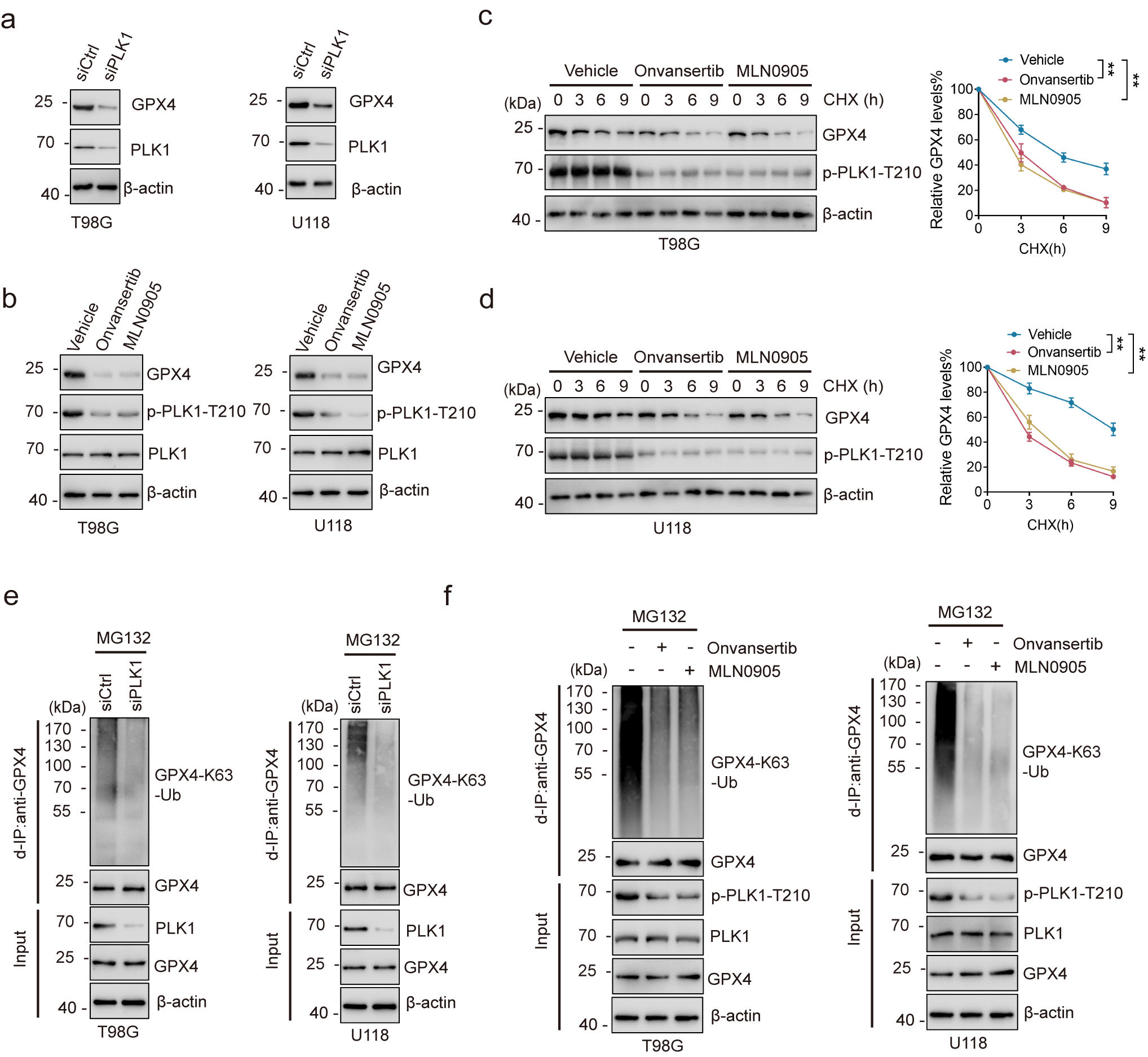


**Fig. S5. PLK1-mediated TRIM26 phosphorylation increases GPX4 stability. a** Western blot assay for GPX4 expression in T98G and U118 cells transfected with control siRNA or PLK1 siRNA. **b** Western blot assay for GPX4 expression in T98G and U118 cells treated with onvansertib (2 nM) or MLN0905 (2 nM) for 4 hours. **c-d** T98G (**c**) and U118 (**d**) cells were treated with onvansertib (2 nM) or MLN0905 (2 nM) and incubated with CHX for the indicated times, the cell lysates were subjected to IB. Quantification of GPX4 levels relative to β-actin is shown. **e** T98G and U118 cells transfected with control siRNA or PLK1 siRNA were subjected to d-IP and IB with the indicated antibodies. **f** After treatment with onvansertib (2 nM) or MLN0905 (2 nM), T98G and U118 cells were subjected to d-IP and IB with the indicated antibodies. Data are represented as the mean ± SD (n=3); student’s t test; **P* < 0.05, ***P* < 0.01, ****P* < 0.001.


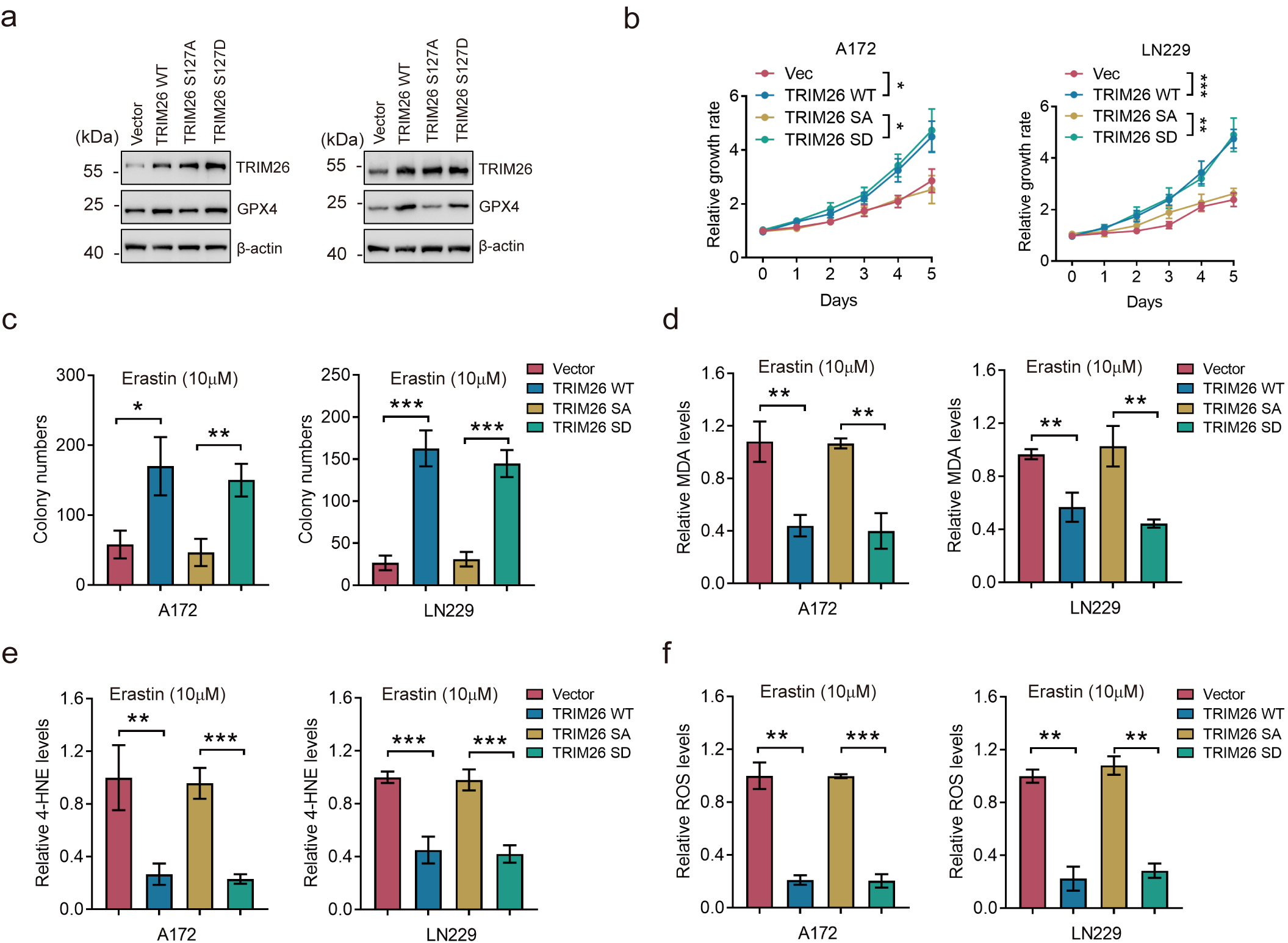


**Fig. S6 S127 phosphorylation is required for TRIM26 function in ferroptosis. a** A172 and LN229 cells transfected as indicated, and the TRIM26 and GPX4 expression levels were determined by IB. **b-c** Cell proliferation of A172 and LN229 cells treated as indicated using CCK-8 assay (**b**) and colony formation assay (**c**). **d-f** Relative MDA (**d**), 4-HNE (**e**), and ROS levels (**f**) in A172 and LN229 cells treated as indicated. Data are represented as mean ± SD (n=3); student’s t test; **P* < 0.05, ***P* < 0.01, ****P* < 0.001.
