# Supplementary material for: The E3 ligase TRIM26 suppresses ferroptosis through catalyzing K63-linked ubiquitination of GPX4 in glioma
Source: Cell Death Dis. 2023 Oct 23;14(10):695. doi: 10.1038/s41419-023-06222-z (PMC10593845; doi:10.1038/s41419-023-06222-z)

Fig. 1a

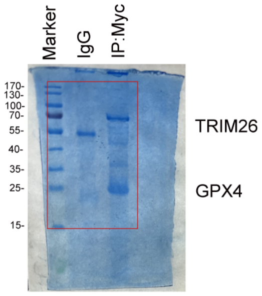

Fig. 1c

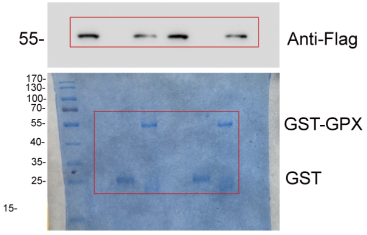

Fig. 1h

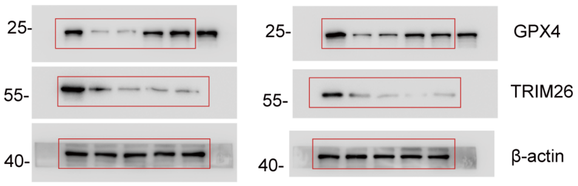

Fig. 1g

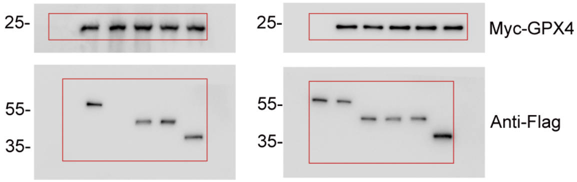

Fig. 1j

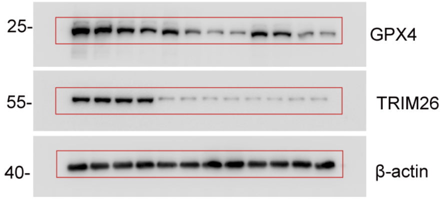

Fig. 1k

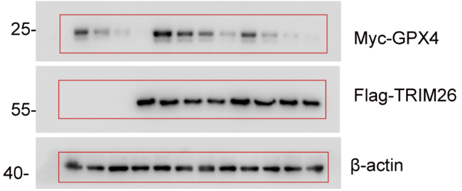

Fig. 2a

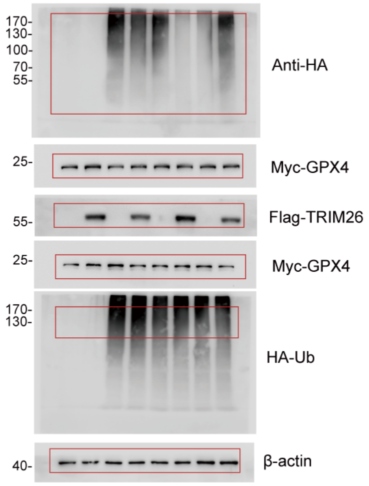

Fig. 2b

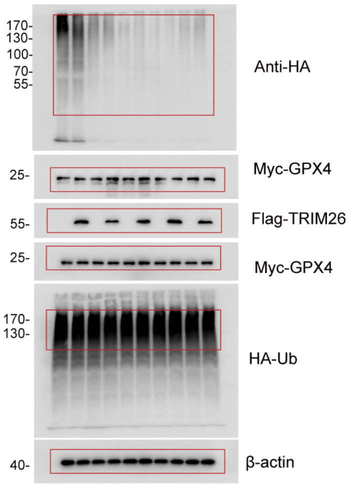

Fig. 2c

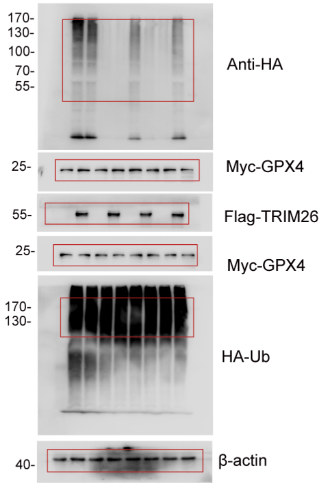

Fig. 2d

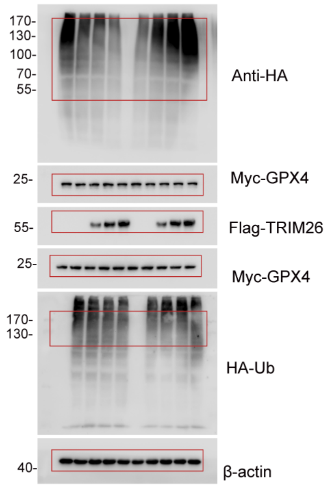

Fig. 2e

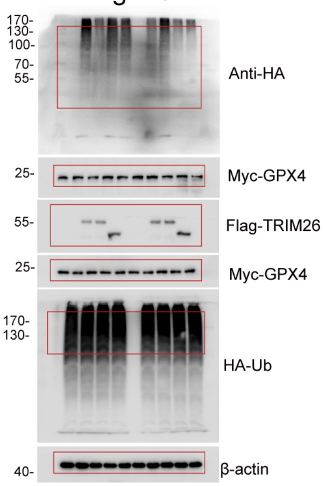

Fig. 2f

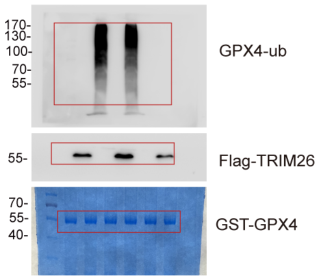

Fig. 2g

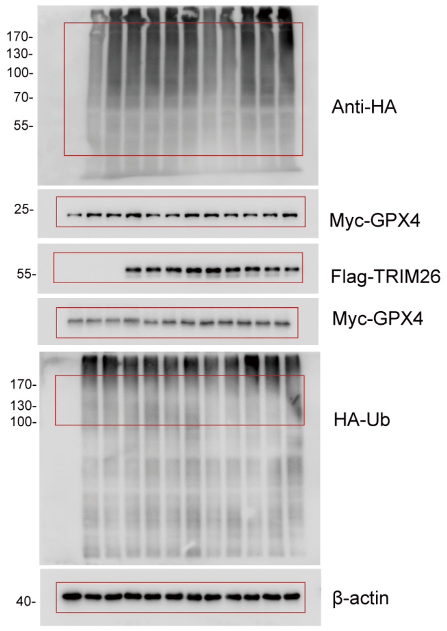

Fig. 2h

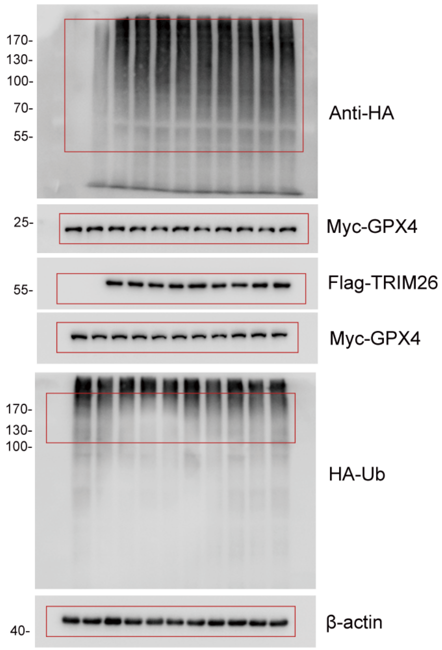

Fig. 2i

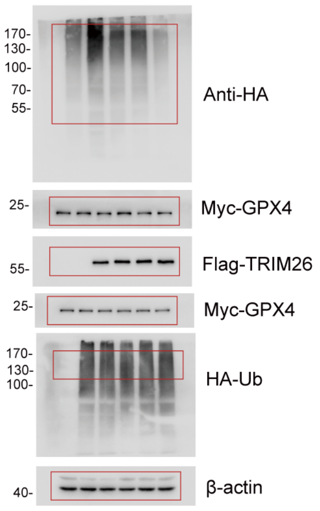

Fig. 4a

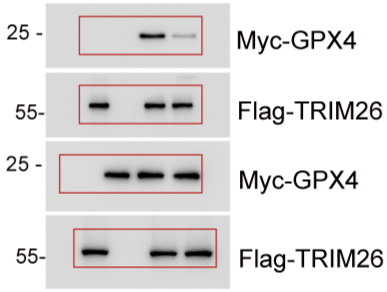

Fig. 4b

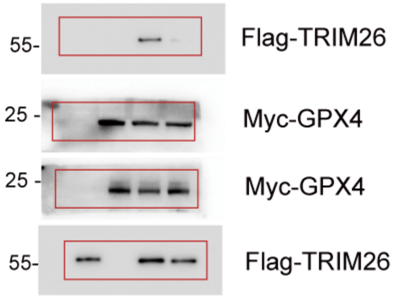

Fig. 4c

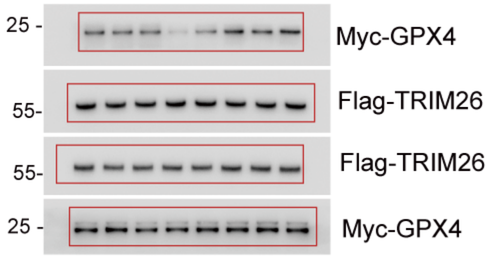

Fig. 4d

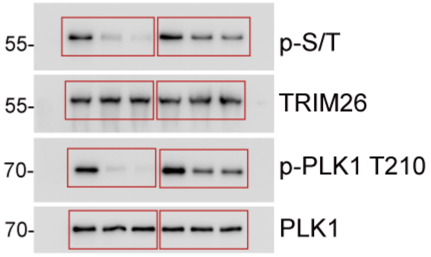

Fig. 4e

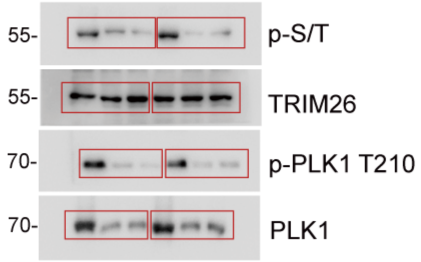

Fig. 4f

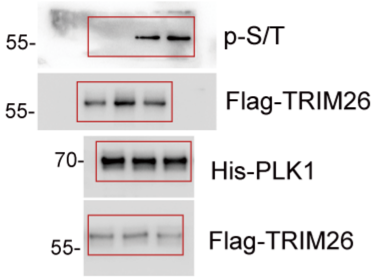

Fig. 4g

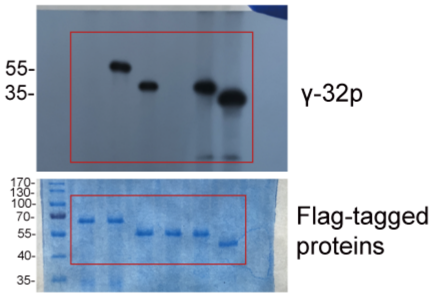

Fig. 4h

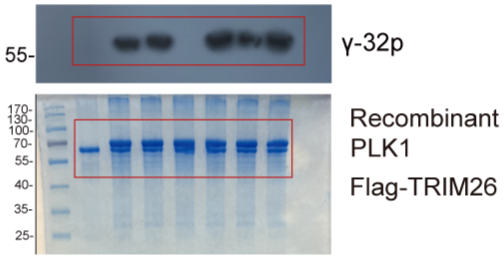

Fig. 5a

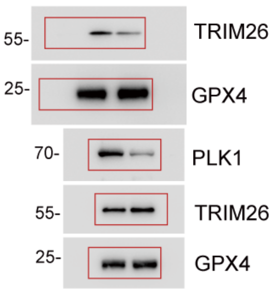

Fig. 5b

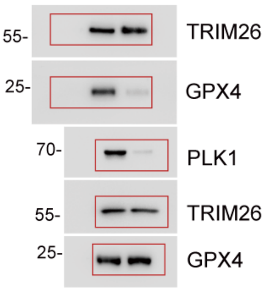

Fig. 5c

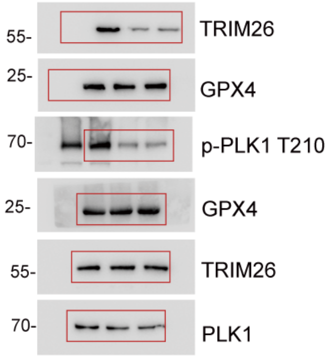

Fig. 5d

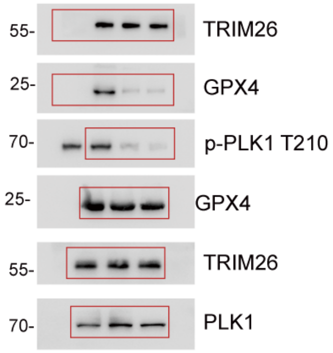

Fig. 5f

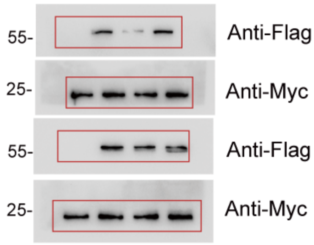

Fig. 5g

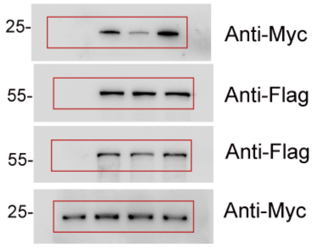

Fig. 5h

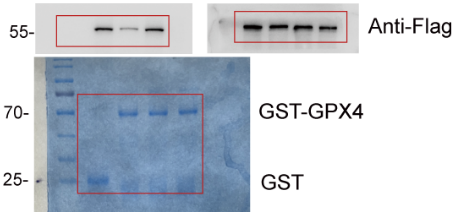

Fig. 5i

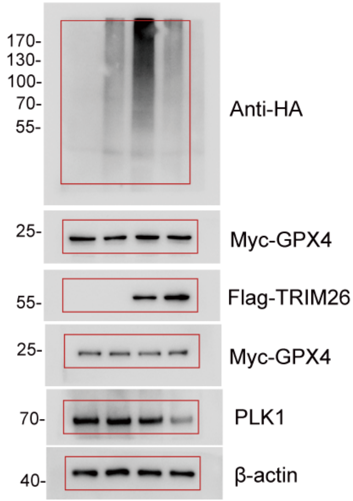

Fig. 5j

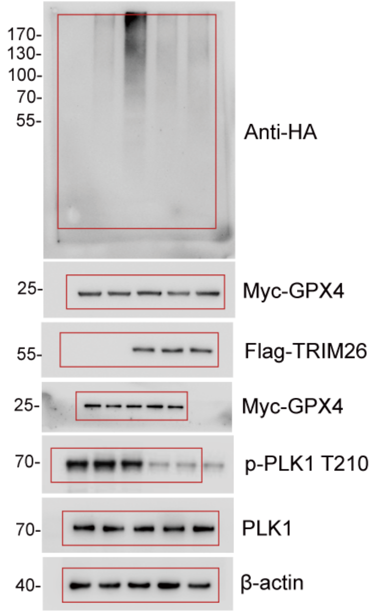

Fig. 5k

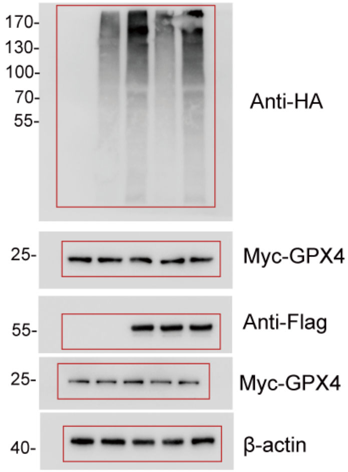

Fig. 5l

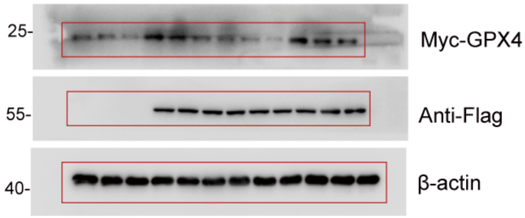

Fig. 6a

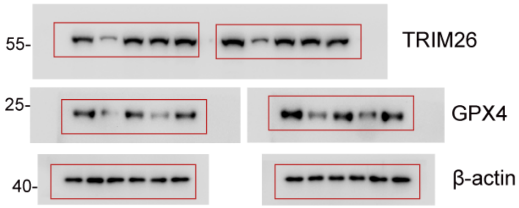

Fig. S1a

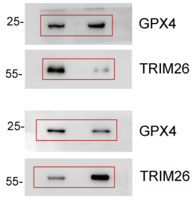

Fig. S1b

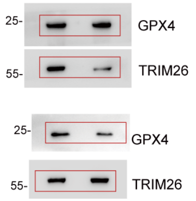

Fig. S1c

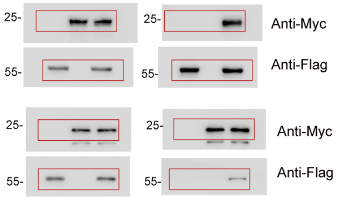

Fig. S1d

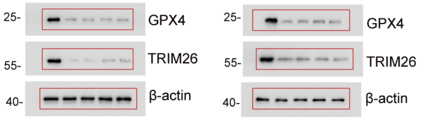

Fig. S1e

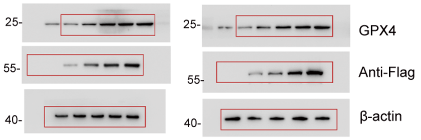

Fig. S1g

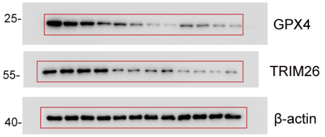

Fig. S1h

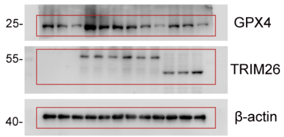

Fig. S1i

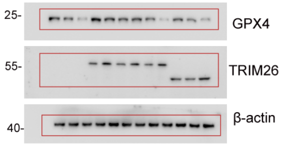

Fig. S2a

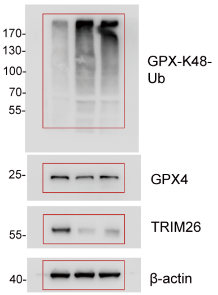

Fig. S2b

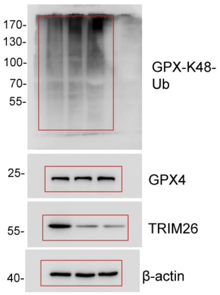

Fig. S2c

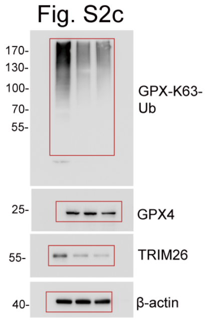

Fig. S2d

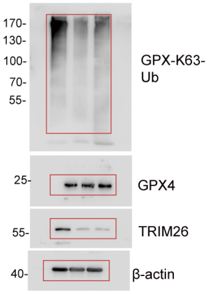

Fig. S3a

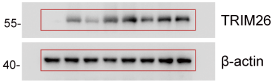

Fig. S3d

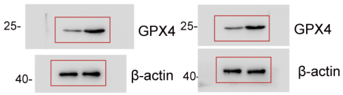

Fig. S3e

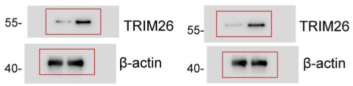

Fig. S4a

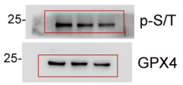

Fig. S4b

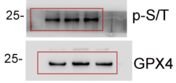

Fig. S4c

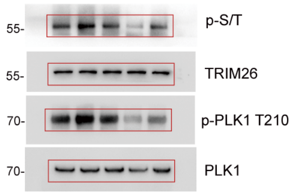

Fig. S4d

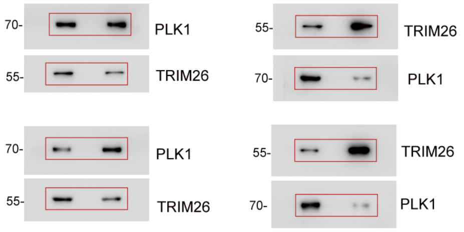

Fig. S4e

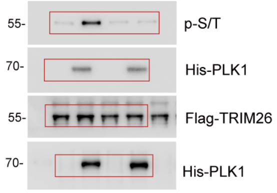

Fig. S5a

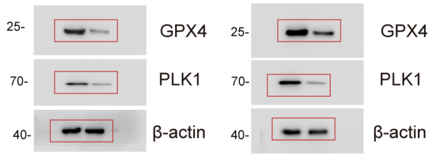

Fig. S5b

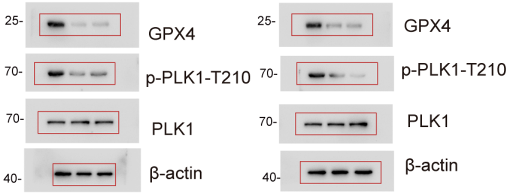

Fig. S5c

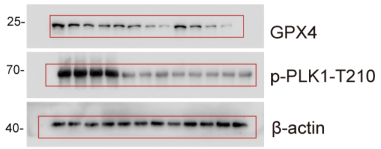

Fig. S5d

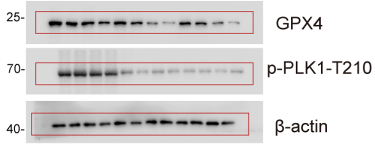

Fig. S5e

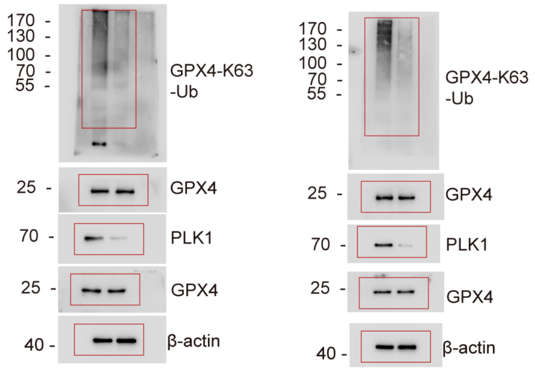

Fig. S5f

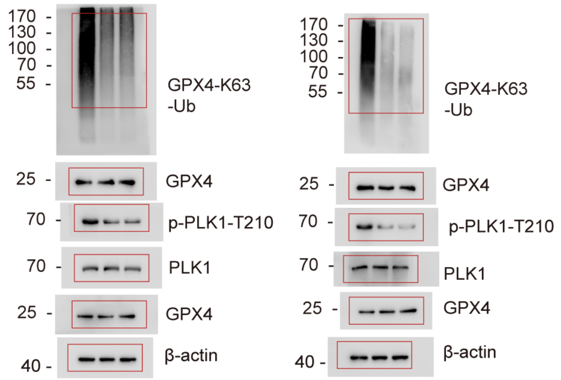

Fig. S6a

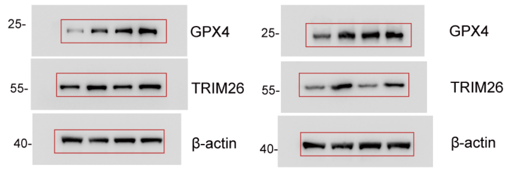

Supplement: Supplementary file 3 — Original Data FileOriginal images of western blot [file 41419_2023_6222_MOESM3_ESM.pdf]
